# Rational Design of Dual-Atom Catalysts for Electrochemical CO_2_ Reduction to C_1_ and C_2_ Products with High Activity and Selectivity: A Density Functional Theory Study

**DOI:** 10.1021/acs.iecr.4c04831

**Published:** 2025-02-18

**Authors:** Zhongze Bai, Zhuo Zhi, Xi Zhuo Jiang, Kai H. Luo

**Affiliations:** † Department of Mechanical Engineering, University College London, Torrington Place, London WC1E 7JE, U.K.; ‡ Department of Electronic and Electrical Engineering, University College London, Torrington Place, London WC1E 7JE, U.K.; § School of Mechanical Engineering and Automation, Northeastern University, Shenyang, Liaoning 110819, PR China

## Abstract

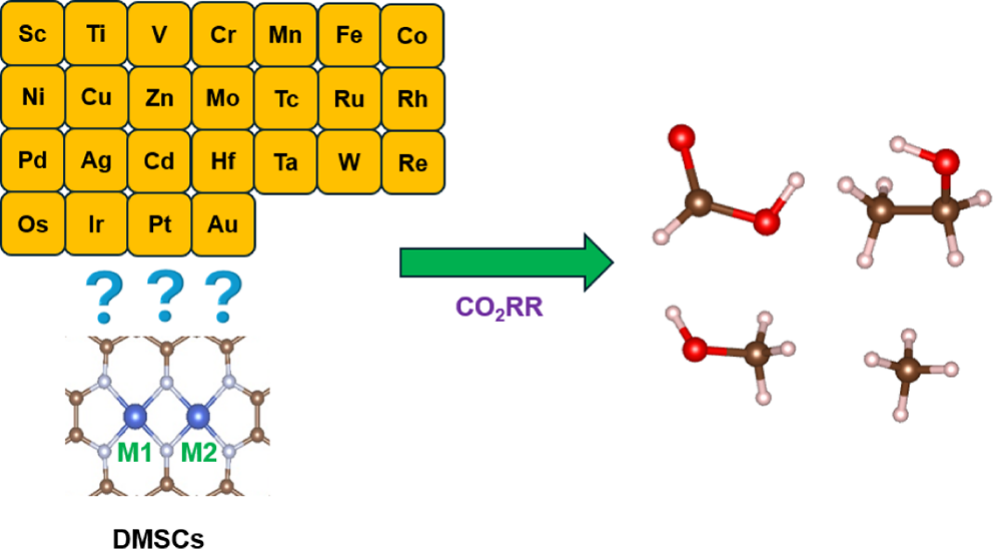

Carbon dioxide (CO_2_) electroreduction using renewable energy provides a sustainable solution to mitigate greenhouse effects and achieve carbon neutrality. Developing high-performance electrocatalysts for the CO_2_ reduction reaction (CO_2_RR) is key to promoting such a technology. Herein, we systematically explored the CO_2_RR catalytic activity of 325 dual-metal-site catalysts (DMSCs) through density functional theory (DFT) calculations. Among them, the Sc/Tc DMSC is particularly advantageous for HCOOH, CH_4_, and CH_3_CH_2_OH production, with limiting potentials of −0.45 V, −0.45 V, and −0.46 V, respectively. The Ti/Rh DMSC can selectively convert CO_2_ to CH_3_CH_2_OH at ultralow overpotentials (*U*_L_ = −0.21 V). HCOOH is the preferred product of the CO_2_RR on the Mn/Fe site with a *U*_L_ of −0.30 V. Mn/Fe presents the highest inhibitory effects on the side reaction, the hydrogen evolution reaction (HER), with a *U*_L_ of −0.66 V. Moreover, electronic analysis was conducted to further explain the enhancement for the CO_2_RR of explored catalysts at the subatomic level. Our work offers a strategy for screening of high-performance DMSCs and reveals the mechanisms of the CO_2_RR to target products for selected catalysts, benefiting the further development of CO_2_RR electrocatalysts.

## 1 Introduction

The overuse of fossil energy has resulted in a significant rise in anthropogenic carbon dioxide (CO_2_) concentration in the atmosphere, which is regarded as one of the primary drivers of global warming.^1−4^ Thus, developing CO_2_ utilization technologies is important and necessary to effectively address climate change and achieve carbon neutrality. The electrochemical CO_2_ reduction reaction (CO_2_RR) by clean renewable energy (sun, wind, etc.) is one of the promising technologies to convert CO_2_ to chemicals.^5−10^ However, the widespread deployment of the electrochemical CO_2_RR is hindered by the unsatisfactory product selectivity, high overpotential, and sluggish kinetics of C_2_ products with higher commercial value.^11−13^ In the CO_2_ electroreduction process, the CO_2_ electroreduction performance is determined by electrocatalysts, and materials such as nonmetals,^14,15^ nanostructured materials,^16−18^ bulk metals, and alloys^19−21^ have been identified as working electrocatalysts.

Recently, dual-metal-site catalysts (DMSCs) hold significant promise for the CO_2_RR and have attracted a lot of research interest from both experiments and simulations. Li and co-workers discovered and synthesized Fe/Ni DMSC, which could promote the CO_2_RR to CO with a *U*_L_ of −0.34 V.^22^ Hao and co-workers found that the Ni/Ni DMSC had a promoting effect on the generation of CO during the CO_2_RR with a Faradaic efficiency of over 99%.^23^ Ag/Ag DMSC was prepared for the CO_2_RR to CO by Li and co-workers by experiments.^24^ They found that the Faradaic efficiency was up to 93.4% and the Ag/Ag catalyst had outstanding stability of more than 36 h.^24^ Bai and co-workers chose the metal Cu, which has been proven to have the ability to reduce CO_2_ to valuable hydrocarbons, as the candidate^25,26^ and investigated the influence of the coordination environment on Cu-SACs and Cu-DACs by density functional theory (DFT) calculations.^27^ The results showed that the CuCuNC-4a DMSC configuration could benefit C_2_ product formation, but it suffered from aggressive competing reactions, hydrogen evolution reaction (HER), and the difficulty of CO_2_ adsorption as well as C_2_ product desorption. Thus, further improvement of the CO_2_RR performance on DMSCs is of great importance and necessity. For DMSCs, the combinations of central metals and nonmetal coordination environments extend the possibility of designing high-performance catalysts for the electrochemical CO_2_RR. High-throughput investigations of DMSCs could greatly contribute to the discovery of high-performance materials. For instance, Luo and co-workers designed Cu/Mn, Ni/Mn, and Ni/Fe DMACs for CO_2_ conversion to CO via screening by DFT calculations.^28^ Li and Tang enhanced the performance of CO generation during the CO_2_RR on M2NC-4a DMSC by changing the central atoms.^29^ Feng and co-workers successfully found four DMSCs for CO_2_ electroreduction to CO through DFT calculations.^30^ Ding and co-workers carried out DFT simulations to investigate the electrochemical CO_2_RR to HCOOH and found five experimentally unexplored DMSCs that presented better CO_2_RR abilities.^31^ Chen and co-workers carried out computational screening for the reduction of CO_2_ to C_2_ products on M2NC–3 DMSCs with six possible transition metal atoms taken into account.^32^ However, those studies mainly considered the adsorption of intermediates on metal atoms and neglected the adsorption of intermediates on surrounding atoms, which may lead to an inadequate evaluation of catalyst properties. Also, there is still a lack of systematic studies on how the central atom of the catalyst affects its behavior in the distribution of both C_1_ and C_2_ products during CO_2_ electroreduction.

**Figure 1:**
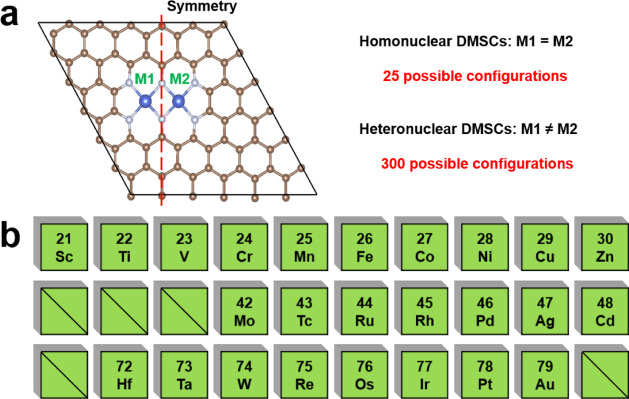
(a) Structures of DMSCs. (b) Screened transition metal elements.

In this study, we systematically screen high-performance DMACs for the generation of both C_1_ and C_2_ chemicals (HCOOH, CH_3_OH, CH_4_, C_2_H_6_O, C_2_H_4_, and C_2_H_6_) during the electrochemical CO_2_RR by DFT calculations. Based on a high-performance DMSC configuration^27^ (Figure 1a), electrocatalysts containing elements from 25 transition metal elements (Sc, Ti, V, Cr, Mn, Fe, Co, Ni, Cu, Zn, Mo, Tc, Ru, Rh, Pd, Ag, Cd, Hf, Ta, W, Re, Os, Ir, Pt, and Au) (Figure 1b) are studied. The full description of the DMSC design and screening procedure is shown in Section 2.2.

## 2 Methods

### 2.1 DFT Calculations

All spin-polarized DFT calculations were carried out using the Vienna Ab initio Simulation Package (VASP).^33,34^ The exchange-correlation interactions were accounted for via the revised Perdew–Burke–Ernzerhof (RPBE) with the generalized gradient approximation (GGA).^35^ The core electrons were treated by the projector augmented-wave (PAW) method.^36^ The DFT-D3 method with Becke–Johnson damping was added to consider van der Waals interactions between atoms.^37,38^ The Monkhorst–Pack *k*-point grids and cutoff energy were set to 3 × 3 × 1 and 500 eV, respectively. A 20 Å vacuum space was added along the *Z*-axis to reduce the interaction between adjacent layers. Convergence criteria of 0.02 eV/Å and 1.0 × 10^–5^ eV were set for the Hellmann–Feynman force and electronic energy, respectively. Implicit solvent models were implemented through the VASPsol code with ϵ_r_ of 78.4 to consider the electrolyte influence during the electrolytic process.^39−42^ During the calculation of intermediate adsorption, we considered all adsorption sites involving both metal and nonmetal atoms and obtained their stable energies, selecting the structure with the lowest energy as the final stable configuration. The Gibbs free energy (Δ*G*) of intermediates throughout the CO_2_ electroreduction process was calculated using the computational hydrogen electrode (CHE) model.^43,44^ In the CHE model, the impact of the applied voltage (*U*) is accounted for by adjusting the free energy of the electrochemical reactions. Specifically, the free energy (Δ*G*) is corrected as:^43^
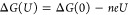
1where, Δ*G*(0) is the free energy under standard conditions, *n* is the number of electrons transferred, *e* is the elementary charge, and *U* is the applied potential.

The influence of pH is incorporated through the concentration of protons (H^+^) involved in proton-coupled reactions. The free energy (Δ*G*) is corrected through eq 2:^43^
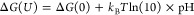
2where, Δ*G*(0) is the free energy under standard conditions, *k*_B_ is the Boltzmann constant, and *T* represents temperature.

To calculate reaction barriers, the climbing image nudged elastic band (CI-NEB) approach was used to search for transition states (TS).^45^ Moreover, VASPKIT^46^ and QVASP^47^ programs were used to aid in the computations. Crystal orbital Hamilton population (COHP) analysis was carried out through the local orbital basis suite toward electronic-structure reconstruction (LOBSTER).^48^ All ab initio molecular dynamics (AIMD) computations were performed at 300 K for 10 ps using a Nose-Hoover thermostat with a time step of 1 fs. The data of the Cu/Cu DMSC are from our previous work.^27^

The adsorption energy (*E*_ads_) was calculated through eq 3:
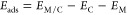
3where *E*_M/C_, *E*_C_, and *E*_M_ denote the total energy of the catalyst and adsorbates, the energy of the catalyst, and the energy of adsorbates, respectively.

The Gibbs free energy (Δ*G*) change was calculated according to eq 4:^49^
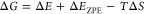
4where Δ*E* is the difference in electron energies obtained directly from DFT calculations. Δ*E*_ZPE_ and Δ*S* are the differences in zero-point energy and entropy between 0 K and room temperature (*T* = 298.15 K), respectively.

The limiting potential (*U*_L_) was calculated by eq 5:^50−52^
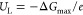
5where Δ*G*_max_ represents the free energy difference between the potential limiting step (PLS) in CO_2_RR at 0 V and the reversible hydrogen electrode (RHE).

To evaluate the thermodynamic stabilities of catalysts, the formation energy (*E*_f_) was adopted and calculated as shown in eq 6:^53^
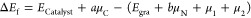
6where *E*_Catalyst_ and *E*_gra_ are the total energies of optimized catalysts and pristine graphene with 72 atoms, respectively. μ_N_ and μ_C_ are the energies of a nitrogen atom and a single carbon atom, respectively, which were calculated from graphene and isolated N_2_ molecules. μ_1_ and μ_2_ are the energies of single metal atoms 1 and 2 in a vacuum, respectively. *a* is the number of C atoms that differ between catalysts and pure graphene. The number of N atoms in catalysts is denoted by *b*.

As shown in eqs 7 and 8,^54^ the binding energy (*E*_bin_) and cohesive energy (*E*_coh_) were calculated to analyze the binding performance between the N-dropped graphene substrate and metal atoms.
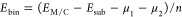
7
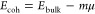
8where *n* and *m* are the number of metal atoms in catalysts and bulk metals, respectively. μ is the single metal atom energy corresponding to the bulk metal.

Energy barrier (*E*_b_) was calculated as presented in eq 9:
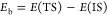
9where *E*(TS) and *E*(IS) are the energies of the transition state and initial state, respectively.

### 2.2 DMSC Screening Procedure

**Figure 2:**
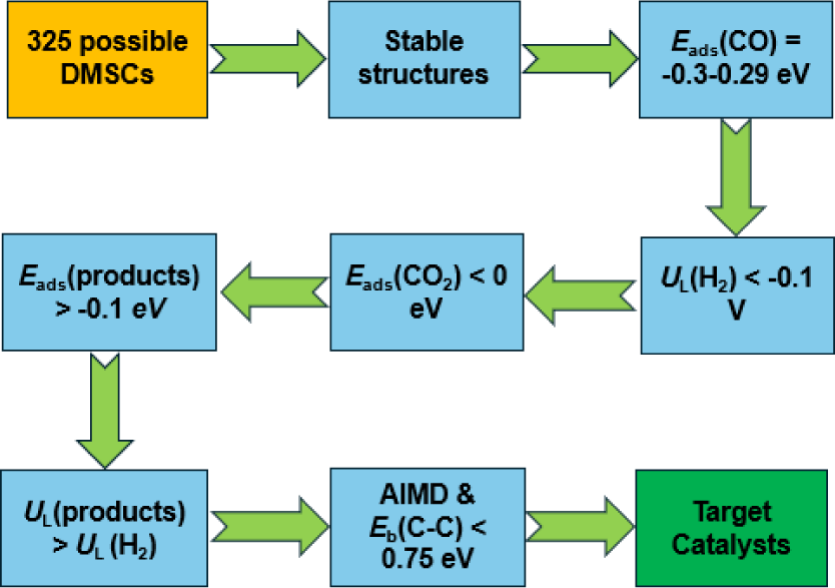
Schematic diagrams for screening DMSCs through the DFT method.

In the DMSC structure, the two metals (M1 and M2) are equivalent sites. A total of 325 possible structures are considered, including 25 homonuclear (M1 = M2) and 300 heteronuclear (M1 ≠ M2) DMSCs. We first screened the stable DMSCs from all 325 possible structures. Second, catalysts with the adsorption energy of CO between −0.3 and 0.29 eV were selected. The competing HER reactions of DMSCs were inhibited by the selection of *U*_L_ (H_2_) lower than −0.1 V. Also, we further screened catalysts with negative adsorption energy of CO_2_, which could benefit the activation of CO_2_ and facilitate the subsequent electrocatalytic reactions. After that, we screened catalysts with adsorption energies of the main C_1_ and C_2_ products higher than −0.1 eV to facilitate the desorption of generated products. Then, we revealed generation mechanisms of target products on DMSCs that satisfy the above conditions and selected catalysts with *U*_L_ of generated chemicals higher than *U*_L_ (H_2_). At last, AIMD simulations at 300 K were carried out to examine the kinetic stabilities of screened catalysts. For C_2_ chemicals, considering C–C coupling is a nonelectroreduction process, the energy barrier of C–C coupling was used as an indicator to determine whether the C_2_ products are readily generated. In detail, catalysts with the energy barrier of C–C coupling less than 0.75 eV could benefit C_2_ product formation, which is widely accepted by previous studies.^32,55^ The screening process of the catalysts is illustrated in Figure 2. More details of the screening criteria are presented in Section 3.

## 3 Results

### 3.1 Identification of DMSCs with High Potential for CO_2_ Reduction

The objective of this part is to identify high-potential DMSCs for further mechanism studies with stable configurations, as well as suitable *E*_ads_(CO), *U*_L_(H_2_), *E*_ads_(CO_2_), and *E*_ads_(products) values.

According to a previous study,^53^ formation energies *E*_f_ and the difference between the binding and cohesive energies *E*_b–c_ (*E*_b–c_ = *E*_bin_ – *E*_coh_) are adopted to evaluate the stability of DMACs. For stable configurations, the values of *E*_f_ and *E*_b–c_ are negative, indicating that catalysts are thermodynamically stable and metal atoms prefer to spread atomically on the graphene rather than aggregate into nanoparticles due to the coordination effect.^27,53^ The objective of this section is to screen out stable DMSCs from the 325 possible configurations by *E*_f_ and *E*_b–c_. The possible combinations of central metals and calculated *E*_f_ and *E*_b–c_ values are shown in Table S1, and 295 stable configurations are found, as shown in Table S2.

CO is a key intermediate for the generation of C_1_ and C_2_ chemicals during the electrochemical CO_2_RR,^56^ and thus the adsorption energy of CO, *E*_ads_(CO), is an important indicator for the screening of DMSCs.^57,58^ A weak CO adsorption indicates that CO is more likely to desorb from the catalyst, forming CO as the final product. Strong CO adsorption will inhibit subsequent reactions, generating valuable feedstocks. Thus, selecting catalysts with moderate *E*_ads_(CO) is important and necessary for the discovery of DMSCs generating hydrocarbons. According to previous studies,^27,59^ the CO adsorption energy of the Cu/Cu DMSC is 0.29 eV; lowering the CO adsorption energy is favorable for the generation of valuable C_1_ and C_2_, however, this value should not be lower than −0.3 eV. The *E*_ads_(CO) values of 295 stable configurations are listed in Table S3, and 46 DMSCs are screened out as shown in Table S4.

During the electrochemical CO_2_RR, the HER is the main competitive reaction. In our previous work, we found that the Cu/Cu DMSC suffered from aggressive competing reactions for H_2_ formation with *U*_L_(H_2_) of only −0.1 V.^27^ To mitigate this problem, we screened out catalysts with *U*_L_(H_2_) values lower than −0.1 V. The *U*_L_(H_2_) values of 46 DMSCs are presented in Table S5. The 37 screened-out catalysts are described in Table S6.

Besides, the adsorption and activation of CO_2_ on the catalyst surface are another obstacle for the CO_2_RR as linear CO_2_ molecules are stable and chemically inert with low electron affinity and a large energy gap.^8,60^ The catalysts with negative adsorption energy of CO_2_ are selected to promote this process.^61^ The calculated *E*_ads_(CO_2_) values are shown in Table S7, and 19 DMSCs are found with negative *E*_ads_(CO_2_) as shown in Table S8.

The desorption of products from catalysts to release the active sites is also an important process affecting the performance of DMSCs. The strong adsorption of products will inhibit the release of target chemicals, and we selected catalysts with adsorption energies of target products above −0.1 eV to ensure that the products could easily detach from the catalyst.^59^ During the CO_2_ electroreduction process, HCOOH, CH_3_OH, and CH_4_ are the key C_1_ chemicals, while C_2_H_4_, C_2_H_6_, and CH_3_CH_2_OH are the main C_2_ products. According to the possible generation mechanisms of target products in our previous work,^27^ the adsorption energies of HCOOH, CH_3_OH, H_2_O&CH_4_, C_2_H_4_, C_2_H_6_, and C_2_H_6_O are used to reflect the generation abilities of HCOOH, CH_3_OH, CH_4_ C_2_H_4_, C_2_H_6_, and C_2_H_6_O, respectively. Table S9 shows the adsorption energies of the main products on 19 selected DMSCs. Overall, we identified a number of catalysts that would favor the generation of HCOOH, CH_3_OH, CH_4_, and C_2_H_6_O. Specifically, Sc/Tc, Sc/Rh, Mn/Fe, Mn/Cd, and Rh/Hf DMSCs can benefit HCOOH formation. Sc/Rh, Mn/Cd, and Rh/Hf DMSCs are favorable for the generation of CH_3_OH. All catalysts except Cu/Pd, Cu/Pt, Pd/Pd, Pd/Pt, and Pt/Pt DMSCs have the potential to promote the formation of CH_4_. Sc/Tc, Sc/Rh, Ti/Cu, Ti/Zn, Ti/Rh, Ti/Hf, Ti/W, Mn/Cd, and Rh/Hf DMSCs favor the C_2_H_6_O desorption.

### 3.2 CO_2_RR Mechanisms and Identification of DMSCs through *U*_L_(products)

After the screening process described above, a total of 16 DMSCs with high potential for C_1_ and C_2_ chemical generation were selected from a total of 325 possible configurations. In this section, we reveal their CO_2_RR mechanisms and calculate their limiting potential for product formation. The possible pathways for C_1_ and C_2_ chemical formation are shown in Figure S1, which refers to earlier studies.^27,62−64^ It should be noted that we only considered the potential pathways for C_2_H_6_O generation for C_2_ compounds, as all screened DMSCs were in favor of the formation of C_2_H_6_O. The most favorable reaction pathways with the highest *U*_L_ values are chosen from the reaction network, and the DMSCs with *U*_L_(products) higher than *U*_L_(H_2_) are screened out for further investigation. After calculating all possible channels for the selected 16 DMSCs, we found that the Sc/Tc DMSC could benefit the generation of HCOOH, CH_4_, and C_2_H_6_O. The Ti/Rh DMSC was screened out for C_2_H_6_O formation. The Mn/Fe DMSC showed good electroreduction catalytic performance on HCOOH generation. The Rh/Hf site was favorable for the formation of HCOOH, CH_3_OH, and C_2_H_6_O during the CO_2_RR. The detailed parameters of the screening process are provided in Table S10. Here, we listed the limiting potentials of the screened DMSCs during the CO_2_RR to target products as well as the Gibbs free energy changes above H_2_ generation during the generation of the products for the discarded catalysts.

#### 3.2.1 CO_2_RR Mechanisms to C_1_ Chemicals

**Figure 3:**
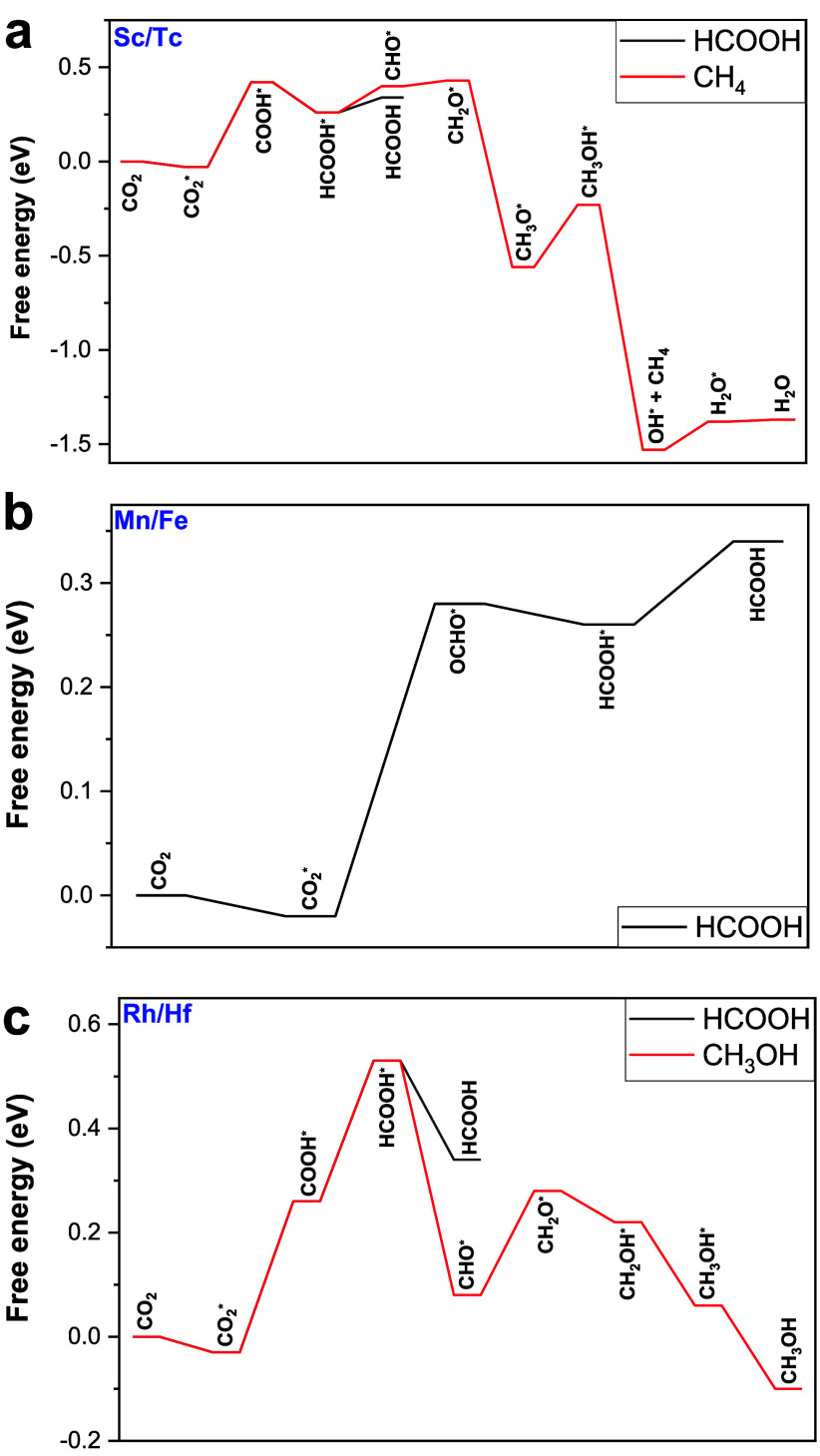
Most favorable reaction pathways of CO_2_ electroreduction to C_1_ chemicals on (a) Sc/Tc, (b) Mn/Fe, and (c) Rh/Hf at 0 applied voltage (symbol * represents the adsorbed state of intermediates).

Figure 3 shows the reaction pathways of electroreduction of CO_2_ to C_1_ chemicals on the Sc/Tc DMSC, Mn/Fe DMSC, and Rh/Hf DMSC. The free energies of elementary steps are listed in Tables S11–S13, respectively. The optimized intermediates for CO_2_ electroreduction on Sc/Tc, Mn/Fe and Rh/Hf sites are presented in Figures S2–S4, respectively. According to Figure 3a, the Sc/Tc DMSC benefits the formation of both HCOOH and CH_4_ via the pathways CO_2_ → CO_2_* → COOH* → HCOOH* → HCOOH and CO_2_ → CO_2_* → COOH* → HCOOH* → CHO* → CH_2_O*→ CH_3_O* → CH_3_OH* → OH* → H_2_O* → H_2_O, respectively. As shown in Figure 3b,c, the pathway for HCOOH formation on the Mn/Fe site is CO_2_ → CO_2_* → OCHO* → HCOOH* → HCOOH. For the Rh/Hf DMSC, the channel is CO_2_ → CO_2_* → OCHO* → HCOOH* → HCOOH. The pathway toward CH_3_OH on the Rh/Hf DMSC is CO_2_ → CO_2_* → COOH* → HCOOH* → CHO* → CH_2_O* → CH_2_OH* → CH_3_OH* → CH_3_OH. CO_2_* → COOH* is the common limiting step for the CO_2_RR on Sc/Tc and Rh/Hf DMSCs with *U*_L_ of −0.45 V and −0.29 V, respectively. For the Mn/Fe site, the conversion of CO_2_* to OCOH* is the limiting step for the conversion of the CO_2_RR to HCOOH with a *U*_L_ of −0.30 V.

#### 3.2.2 CO_2_RR Mechanisms to C_2_ Chemicals

**Figure 4:**
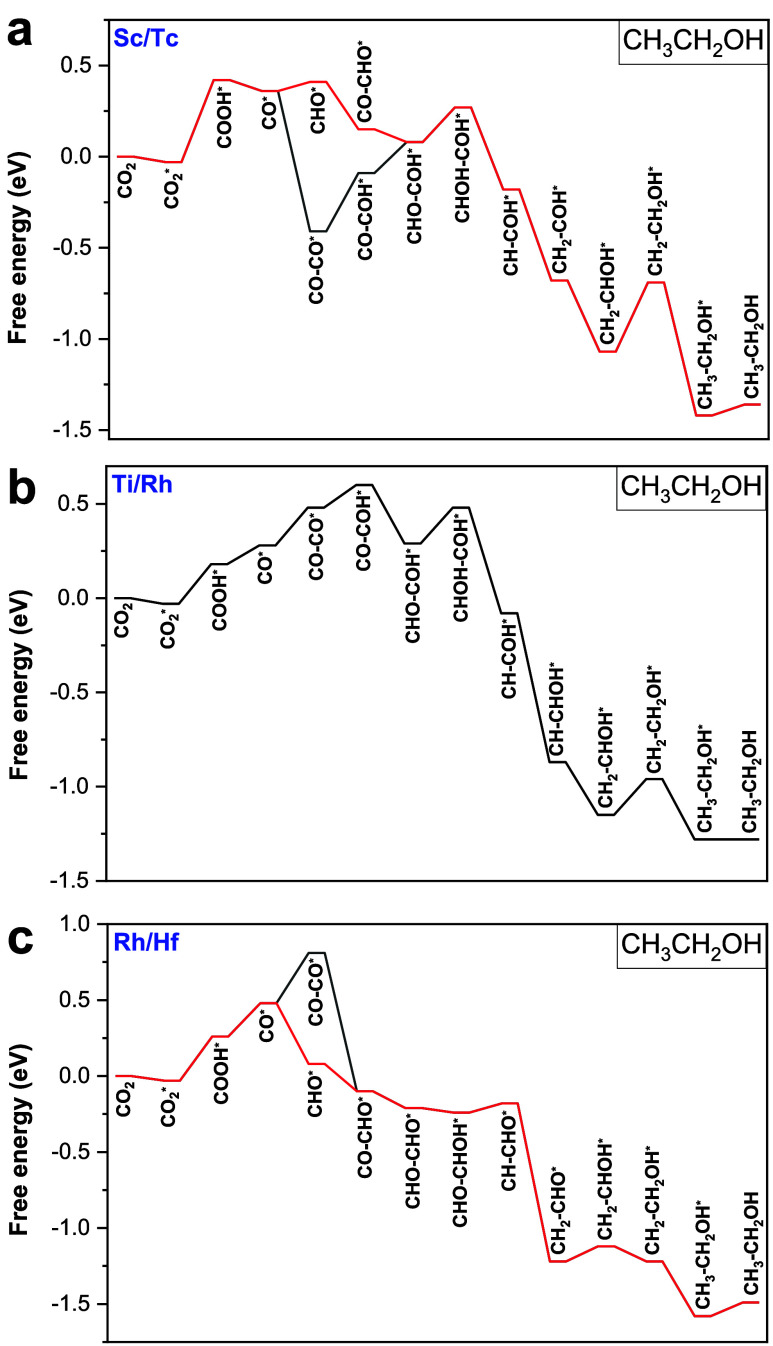
Most favorable reaction pathways of CO_2_ electroreduction to CH_3_CH_2_OH on (a) Sc/Tc, (b) Ti/Rh, and (c) Rh/Hf at 0 applied voltage (symbol * represents the adsorbed state of intermediates).

According to previous studies,^42,65^ CO*–CO*, CO*-CHO*, and CO*-COH* are three main pathways for C–C coupling, which is the key step for C_2_ chemical generation. Among them, CO*-COH* is not considered in the current work as the formation of COH* via CO* → COH* is difficult, with relatively high free energy changes of 1.49, 1.56, and 1.47 eV on Sc/Tc, Ti/Rh, and Rh/Hf DMSCs, respectively. Figure 4 shows the most favorable reaction pathways with the lowest limiting potential of CO_2_ electroreduction to CH_3_CH_2_OH on target DMSCs. The channels for the CO_2_RR to CH_3_CH_2_OH on Sc/Tc, Ti/Rh, and Rh/Hf DMSCs are CO_2_ → CO_2_* → COOH* → CO* → CO–CO*/CHO* → CO–COH*/CO–CHO* → CHO–COH* → CHOH–COH* → CH–COH* → CH_2_–COH* → CH_2_–CHOH* → CH_2_–CH_2_OH* → CH_3_–CH_2_OH* → CH_3_–CH_2_OH, CO_2_ → CO_2_* → COOH* → CO* → CO–CO* → CO–COH* → CHO–COH* → CHOH–COH* → CH–COH* → CH–CHOH* → CH_2_–CHOH* → CH_2_–CH_2_OH* → CH_3_–CH_2_OH* → CH_3_–CH_2_OH and CO_2_ → CO_2_* → COOH* → CO* → CO–CO*/CHO* → CO–CHO* → CHO–CHO* → CHO–CHOH* → CH–CHO* → CH_2_–CHO* → CH_2_–CHOH* → CH_2_–CH_2_OH* → CH_3_–CH_2_OH* → CH_3_–CH_2_OH, respectively. Figures S5–S7 present the optimized intermediates for the CO_2_RR on Sc/Tc, Ti/Rh, and Rh/Hf DMSCs. The relevant free energies of elementary steps are presented in Tables S14–S16, respectively. The limiting step for CH_3_CH_2_OH formation on the Sc/Tc site is CHOH–COH* → CH–COH* with a *U*_L_ of −0.46 V. The *U*_L_ for CH_3_CH_2_OH generation on the Ti/Rh DMSC is −0.21 V via channel CO* to COOH*. CO_2_* → COOH* is the limiting step for the CO_2_RR toward CH_3_CH_2_OH on the Rh/Hf DMSC with a *U*_L_ of −0.29 V.

### 3.3 Kinetic Stability Analysis

To discuss the kinetic stability of the four discovered DMSCs, 10 ps AIMD simulations at 300 K are carried out for Sc/Tc, Ti/Rh, and Mn/Fe DMSCs. For the Rh/Hf site, only 2.44 ps AIMD simulations are carried out, as the Hf atom has detached from the catalyst at the moment, which indicates that this configuration is dynamically unstable and therefore is screened out. Figure S8 presents the geometric structures before and after the AIMD simulations. Although Sc/Tc, Ti/Rh, and Mn/Fe DACs show some changes after AIMD simulations, there is still strong binding between the two metal atoms and their surrounding N atoms, and no evident structural deformation has been identified. Thus, these three potential DACs have a high degree of kinetic stability.

### 3.4 C–C Coupling Process and Performance Comparison of Discovered DMSCs

**Figure 5:**
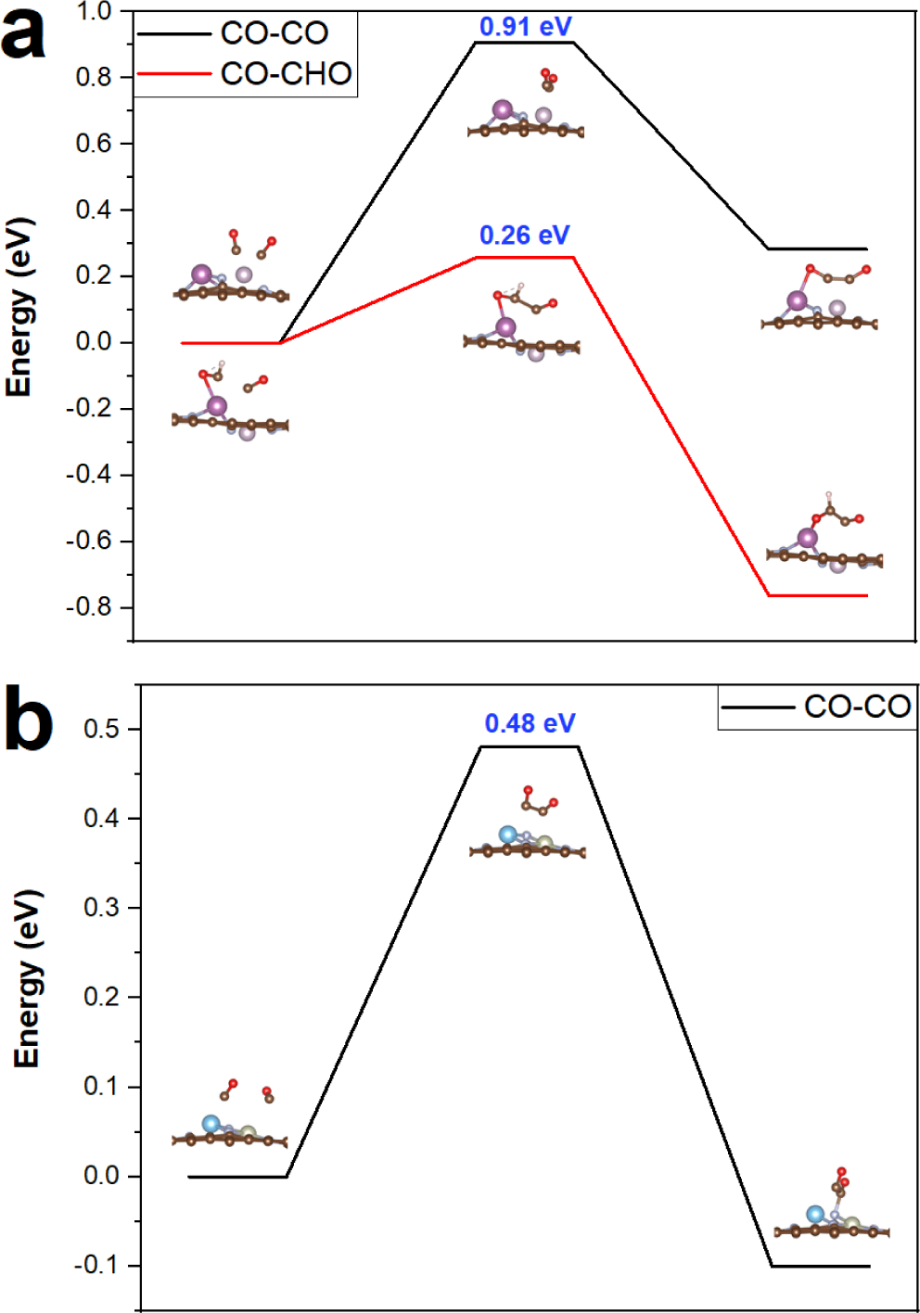
Coupling process of CO–CO*/CO–CHO* on the two possible DMSCs. (a) CO-CO* and CO–CHO* coupling on Sc/Tc, and (b) CO–CO* coupling on Ti/Rh.

According to previous studies, the C–C coupling is a nonelectrochemical process and the energy barrier (*E*_b_) is the important factor in determining whether the C–C bond can form.^32,66,67^ The C–C coupling process on Sc/Tc and Ti/Rh DMSCs is shown in Figure 5. By comparing the threshold value of *E*_b_ of 0.75 eV,^32,55^ the CO–CHO* on Sc/Tc and CO–CO* coupling on Ti/Rh are easy to happen with *E*_b_ of 0.26 and 0.48 eV, respectively. However, the CO–CO coupling on Sc/Tc suffers from a high *E*_b_ of 0.91 eV (higher than 0.75 eV). C–C bond formation on the Sc/Tc DMSC is more favored through CO–CHO coupling with the pathway of CH_3_CH_2_OH formation of CO_2_ → CO_2_* → COOH* → CO* → CHO* → CO–CHO* → CHO–COH* → CHOH–COH* → CH–COH* → CH_2_–COH* → CH_2_–CHOH* → CH_2_–CH_2_OH* → CH_3_–CH_2_OH* → CH_3_–CH_2_OH* → CH_3_–CH_2_OH.

To better clarify the performance of the discovered catalysts, we compared the *U*_L_ and *E*_ads_ of the main chemicals during the CO_2_RR on Cu/Cu, Sc/Tc, Ti/Rh, and Mn/Fe DMSCs, as shown in Table 1. Compared with the Cu/Cu DMSC, all three discovered DMSCs present strong inhibitory and promotional effects on H_2_ production and CO_2_ adsorption, respectively. Among them, the Sc/Tc DMSC has the most pronounced inhibition of H_2_ with its *U*_L_ as low as −0.66 V. There was no significant difference in the promotion of CO_2_ adsorption among the four DMSCs. They all have higher product selectivity than the Cu/Cu DMSC with *U*_L_ (products) less than *U*_L_ (H_2_). The Ti/Rh DMSC requires the lowest external voltage with *U*_L_ (products) of only −0.21 V and consumes the least amount of energy to generate the product in the electrocatalytic process. Regarding product desorption, Sc/Tc and Ti/Rh DMSCs greatly improve C_2_H_6_O desorption compared to the Cu/Cu site.

**Table 1:** Summary of *U*_L_ and *E*_ads_ of the Main Chemicals during the CO_2_RR on Cu/Cu, Sc/Tc, Ti/Rh, Mn/Fe, and Rh/Hf DMSCs^a^

*U*_L_ (V)	Cu/Cu	Sc/Tc	Ti/Rh	Mn/Fe	*E*_ads_ (eV)	Cu/Cu	Sc/Tc	Ti/Rh	Mn/Fe
H_2_	–0.10	–0.66	–0.43	–0.47	CO_2_	0.18	–0.03	–0.03	–0.02
HCOOH	–0.10	–0.45		–0.30	HCOOH	0.03	–0.08		–0.08
CH_3_OH	–0.51				CH_3_OH	–0.05			
CH_4_	–0.51	–0.45			CH_4_	0.23	0.00		
C_2_H_6_O	–0.36	–0.46	–0.21		C_2_H_6_O	–0.19	0.01	0.00	

a The data for the Cu/Cu DMSC are reproduced from ref (27) available under a CC-BY 3.0 license. Copyright 2023 Bai, Z.; Jiang, X. Z.; and Luo, K. H.

### 3.5 Electronic Analysis

Based on the above analysis, the CO_2_RR performance of DMSCs is significantly enhanced by altering the central metal atoms, specifically inhibiting H_2_ generation as well as promoting CO_2_ adsorption. To further clarify the enhancement of the CO_2_RR for the explored catalysts, the Sc/Tc DMSC is selected as the representative that has the lowest *U*_L_ (CO_2_) and *U*_L_ (H_2_) among explored catalysts for electronic analysis through charge density difference (CDD), partial density of states (PDOS) and crystal orbital Hamilton population (COHP) analysis.

**Figure 6:**
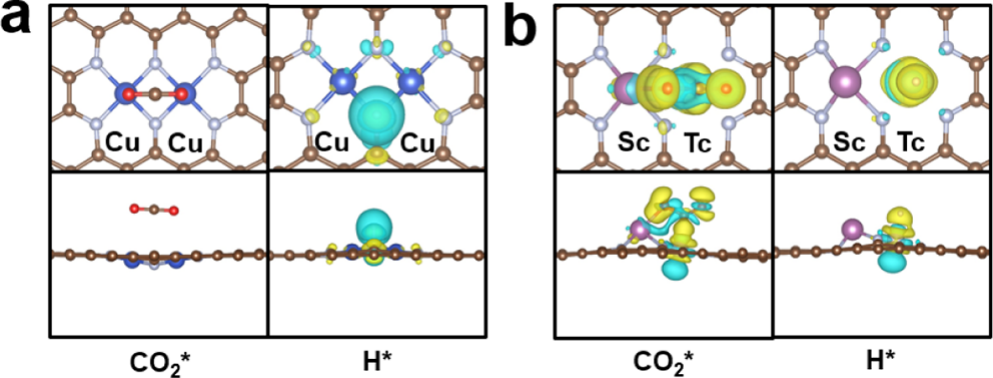
Charge density difference (CDD) of adsorbed CO_2_ and H on the (a) Cu/Cu DMSC and (b) Rh/Hf DMSC. The isosurface value is 0.004 e/bohr^3^. Yellow and blue regions denote charge accumulation and depletion, respectively.

Figure 6a,b present the CDD of adsorbed CO_2_ and H on Cu/Cu and Sc/Tc DMSCs, respectively. The position numbers of C, N, O, as well as H and central metal atoms are shown in Figure S9, and the relevant Bader charges are listed in Table S16. There was no significant charge transfer during the adsorption of CO_2_ on the Cu/Cu DMSC, with a total charge transfer of only 0.02 electrons. For the Sc/Tc catalyst, electrons transfer from the catalyst to CO_2_ during the adsorption process, with a 0.90 charge change through Tc–C and Sc–O1 bonds. CO_2_ adsorbs onto catalyst Sc/Tc with significantly more charge transfer than the Cu/Cu DMSC, indicating that the Sc/Tc site adsorbs CO_2_ more strongly than the Cu/Cu DMSC and thus has a lower adsorption energy. For the H atom, H adsorbs to the Cu/Cu site by generating the N2–H bond with the N2 atom, and 0.48 electrons are transferred from the H atom to the N2 atom. However, H forms a Tc–H bond with the Tc atom on the Sc/Tc DMSC, with 0.20 electrons (less than that of the Cu/Cu DMSC) transferring from the Tc atom to the H atom. The bond length of Tc–C (1.62 Å) is longer than that of N2–C (1.02 Å), indicating the Cu/Cu site adsorbs H more strongly than the Sc/Tc DMSC, with adsorption energies of −0.10 and 0.66 eV, respectively.

**Figure 7:**
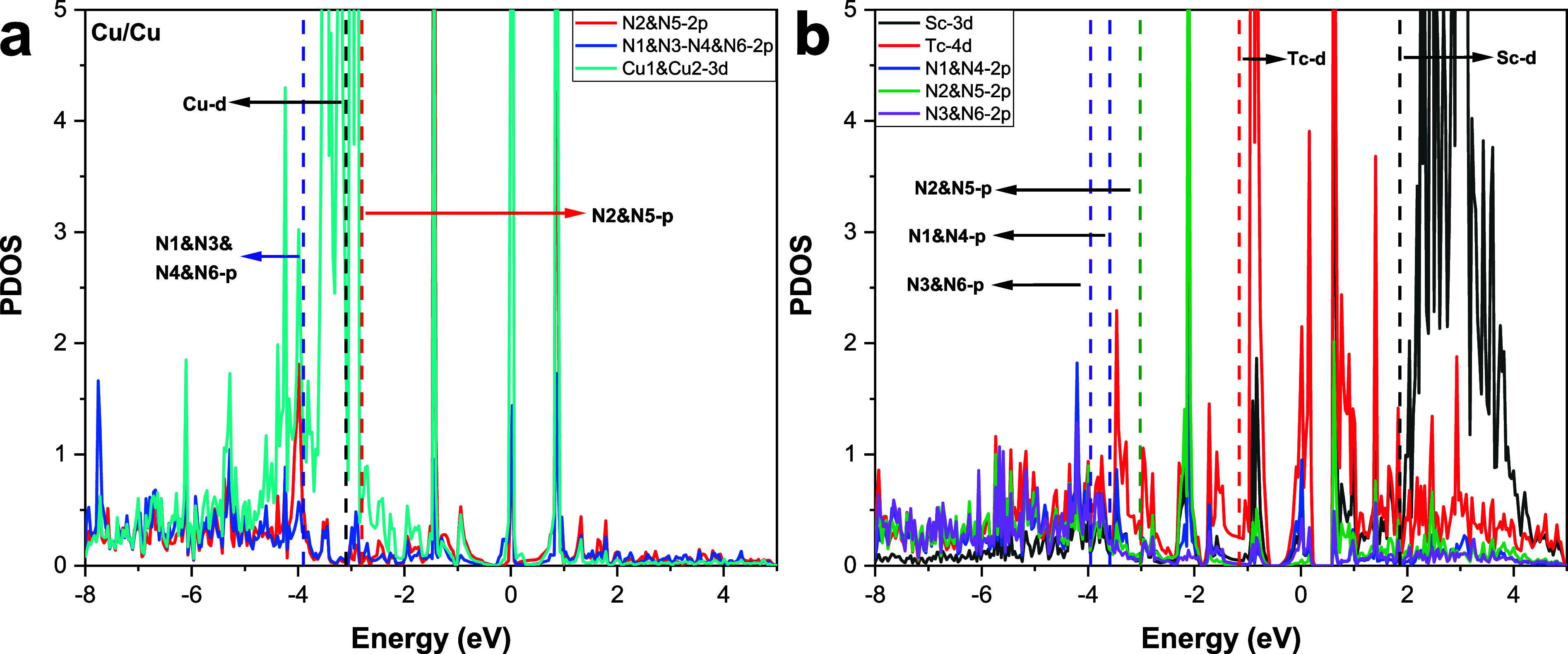
Partial density of states (PDOS) analysis of the (a) Cu/Cu DMSC (reproduced from ref (27), available under a CC-BY 3.0 license. Copyright 2023, Bai, Z.; Jiang, X. Z.; and Luo, K. H) and (b) Sc/Tc DMSC. The dotted lines in the diagram represent the bond centers of atoms.

Figure 7a,b display the partial density of states (PDOS) results of Cu/Cu and Sc/Tc DMSCs. Bond centers of relevant atoms are listed in Table S17. For the Cu/Cu site, PDOS values of N1, N3, N4, and N6 are the same, and the 2p bond center is −3.9 eV. The Cu-3d bond center is −3.1 eV, and the 2p bond centers of both N2 and N5 are −2.8 eV. Among them, the 2p bond center of N2 and N5 is the closest to the Fermi energy level (0 eV), which means N2 and N5 sites have a stronger interaction with intermediates. This agrees well with the adsorption sites for H and other important carbon-containing intermediates on the Cu/Cu DMSC according to our previous research.^27^ However, the reason why the sites of CO_2_ adsorption are not on N2 and N5 atoms could be due to the strong attraction of N atoms to charge and CO_2_ will lose electrons in the adsorption process, which is the opposite of CO_2_ adsorption and bending activation (getting electrons). The poor stability of the CO_2_ intermediate after losing electrons makes the system relatively unstable. Thus, the active site for the most stable adsorption of CO_2_ changes from N2 and N5 to Cu1 and Cu2, where CO_2_ is more accessible to electrons. Regarding the Sc/Tc DMSC, the bond centers of Sc-3d and Tc-4d are 1.9 eV and −1.2 eV, respectively. The 2p bond centers of N1&N4, N2&N5, and N3&N6 are −3.6 eV, −3.0 eV, and −4.0 eV, respectively. As Tc-4d’s bond center is the closest to the Fermi energy level among those atoms, Tc has a stronger interaction with intermediates, which is consistent with the observation in Figure 6b that CO_2_ and H are adsorbed on the Tc atom of Sc/Tc DMSC.

**Figure 8:**
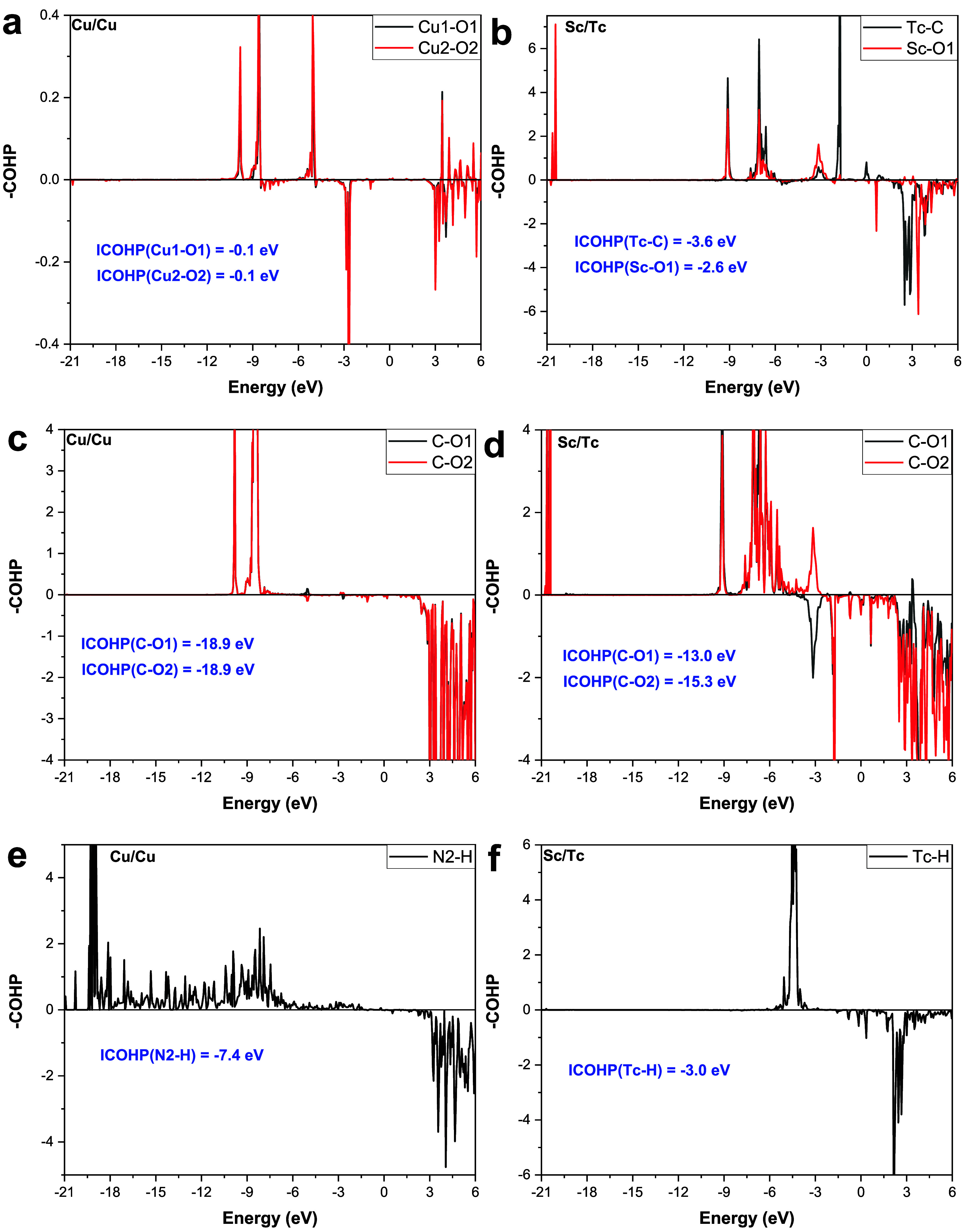
Crystal orbital Hamilton population (COHP) between Cu1–O1 and Cu2–O2 on (a) the Cu/Cu DMSC, Tc–C and ScO1 on (b) the Sc/Tc DMSC, C–O1 and C–O2 on (c) the Cu/Cu DMSC, C–O1 and C–O2 on (d) the Sc/Tc DMSC, N2–H on (e) the Cu/Cu DMSC, and (f) Tc–H on the Sc/Tc DMSC.

COHP calculations are carried out during CO_2_ and H adsorption on Cu/Cu and Sc/Tc catalysts for quantitative evaluation of the strength of different chemical bonds, as shown in Figure 8. During CO_2_ adsorption onto the Cu/Cu DMSC, the −CHOPs of Cu1–O1&Cu2–O2 (Figure 8a) and C–O1&C–O2 (Figure 8c) bonds are the same, with integrated COHP (ICOHP) values of −0.1 eV and −18.9 eV, respectively. For CO_2_ adsorption on the Sc/Tc site, the ICOHP values of Tc–C and Sc–O1 bonds are −3.6 eV and −2.6 eV (Figure 8b), which are lower than those of Cu1–O1 and Cu2–O2, corresponding to lower CO_2_ adsorption energy, again demonstrating the stronger adsorption abilities of Sc/Tc to CO_2_. The values of ICOHP for C–O1 and C–O2 are −13.0 eV and −15.3 eV (Figure 8d), respectively, which are higher than those of C–O1&C–O2. This implies that CO_2_ adsorbs on Sc/Tc with lower C–O bond energies, and Sc/Tc promotes CO_2_ activation, which also proves that low CO_2_ adsorption energies facilitate CO_2_ activation for subsequent reactions. Regarding H adsorption, according to Figure 8e,f, the ICOHP values of N2–H and Tc–H are −7.4 eV and −3.0 eV, respectively. Due to the lower ICOHP value for the Tc–H bond, the adsorption energy of H on the Sc/Tc catalyst will be higher, which in turn inhibits the H_2_ generation reaction.

## 4 Conclusions

In the present study, we systematically explore the catalytic activity and selectivity of dual-metal-site catalysts (DMSCs) considering all of the central metals and the environmental nonmetals through DFT calculations. Considering stabilities, adsorption energy of CO, limiting potential of H_2_, adsorption energy of CO_2_, energy change of CO–CO, adsorption energy of main chemicals, limiting potential of target products and energy barriers of the C–C coupling process, three novel high-performance DMSCs with high activity and selectivity for product formation during the CO_2_RR from 325 possible structures are discovered. Specifically, the Sc/Tc DMSC is favorable for HCOOH, CH_4_, and CH_3_CH_2_OH formation with limiting potentials of −0.45 V, −0.45 V, and −0.46 V, respectively. The Ti/Rh DMSC can selectively reduce CO_2_ to CH_3_CH_2_OH with ultralow overpotentials (*U*_L_ = −0.21 V). HCOOH is the target product for the Mn/Fe site with a *U*_L_ of −0.30 V. Mn/Fe has the most significant inhibitory performance on the HER among those catalysts with a *U*_L_ of −0.66 V. Our study sheds light on the CO_2_RR mechanism of DMSCs and proves that constructing heteronuclear dual-atom active sites is an effective method to develop high-performance electrocatalysts. Finally, we establish a systematic procedure to explore outstanding catalysts for the CO_2_RR. Future work could involve experimental validation and the industrial application of the high-performance catalysts identified in this study. Additionally, exploring the integration of DFT with machine learning could further reduce computational costs and accelerate the design of new catalysts.

## Supporting Information Available

CO_2_RR pathways (Figure S1); optimized substrate and intermediate structures (Figures S2–S7); AIMD simulations (Figure S8); atomic number (Figure S9); catalyst composition during screening (Tables S1–S10); free energy for elementary steps (Tables S11–S16); bond center analysis (Table S17) (PDF)
