# Supplementary material for: Rational Design of Dual-Atom Catalysts for Electrochemical CO2 Reduction to C1 and C2 Products with High Activity and Selectivity: A Density Functional Theory Study
Source: Ind Eng Chem Res. 2025 Feb 18;64(8):4378–87. doi: 10.1021/acs.iecr.4c04831 (PMC11869162; doi:10.1021/acs.iecr.4c04831)
Supplement: Supplementary file 1 — ie4c04831_si_001.pdf [file ie4c04831_si_001.pdf]

Supporting Information

Rational design of dual-atom catalysts for electrochemical CO_2 reduction to C_1 and C_2 products with high activity and selectivity: a density functional theory study

Zhongze Bai[1], Zhuo Zhi[2], Xi Zhuo Jiang[3]*, Kai H. Luo[1]*

[1]Department of Mechanical Engineering, University College London,

Torrington Place, London, WC1E 7JE, UK

[2]Department of Electronic and Electrical Engineering, University College London,
Torrington Place, London, WC1E 7JE, UK

[3]School of Mechanical Engineering and Automation, Northeastern University,
Shenyang, Liaoning, 110819, PR China
* Corresponding Author
Email: jiangxz@mail.neu.edu.cn & k.luo@ucl.ac.uk

Supplementary Figures

a

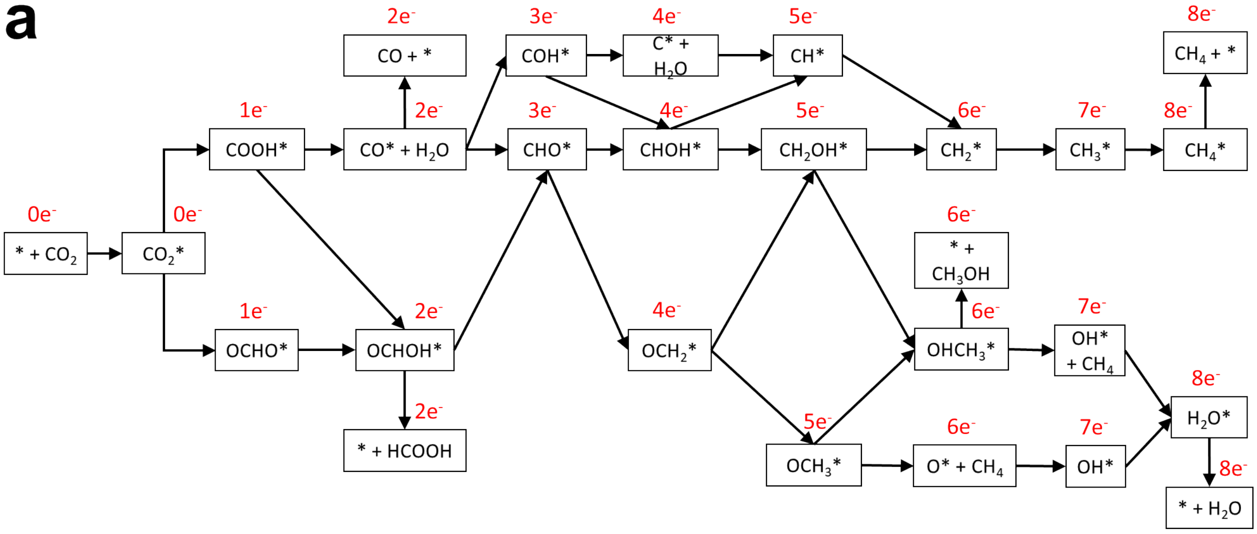

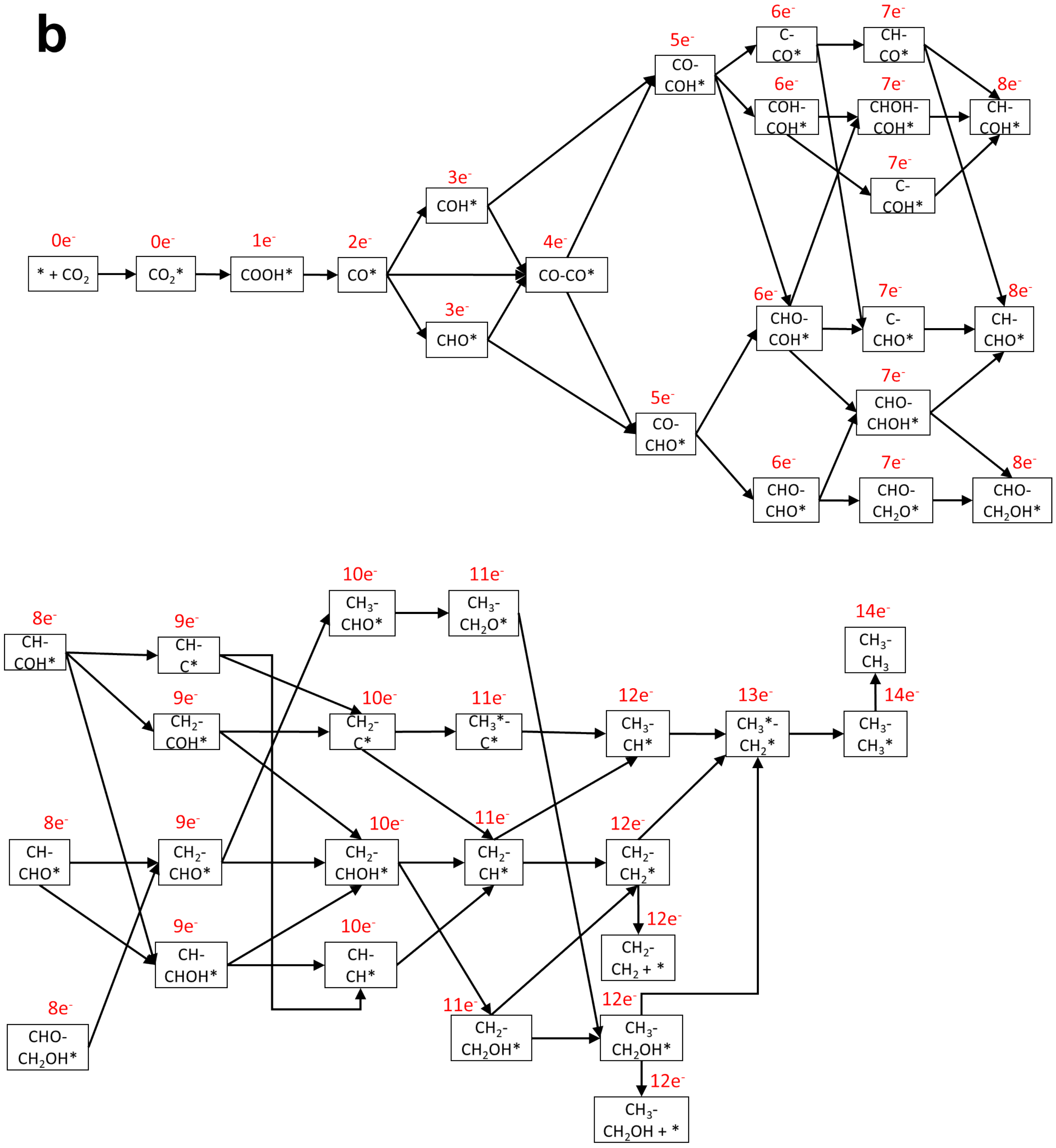

Figure S1 Possible pathways for CO_2 electroreduction to (a) C_1 and (b) C_2 products on DMSCs (symbol * presents adsorbed state of intermediates). Reproduced from ref [1]. Available under a CC-BY 3.0 license. Copyright 2023 Bai, Z.; Jiang, X. Z.; and Luo, K. H.

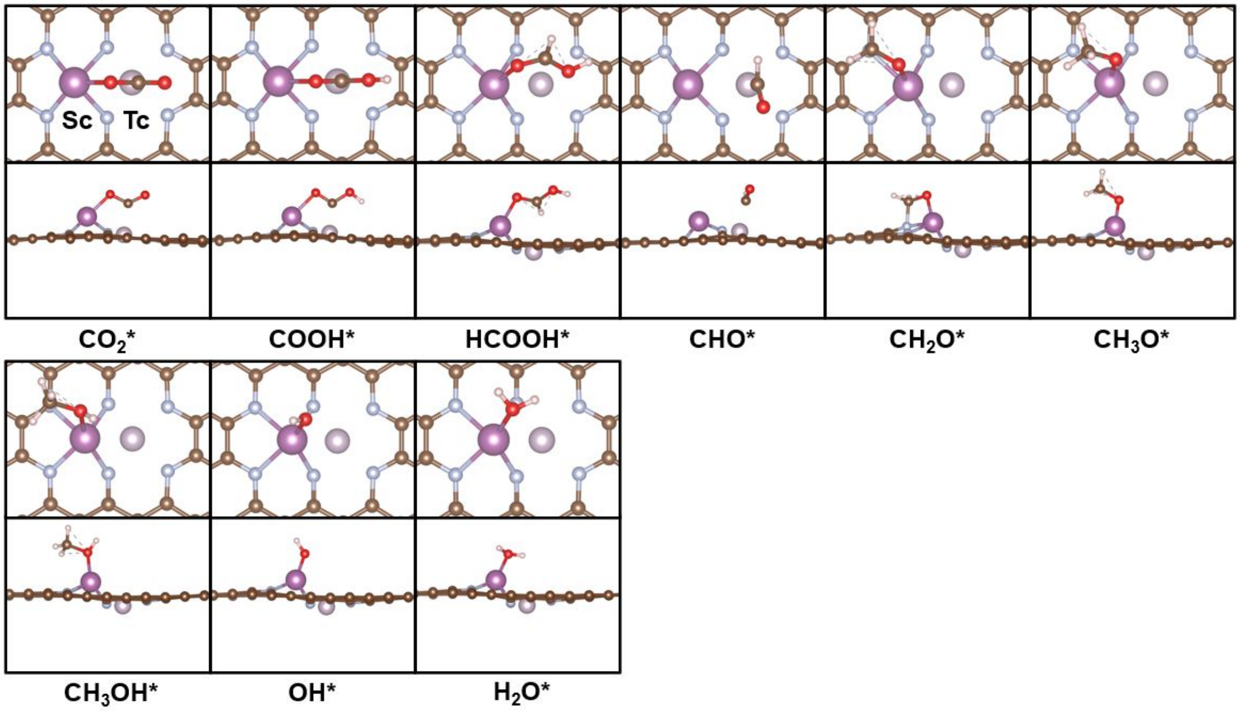

Figure S2 Optimized intermediates for CO_2 electroreduction to HCOOH and CH_4 on Mn/Fe DMSC site (brown: C; gray: N; red: O; white: H).

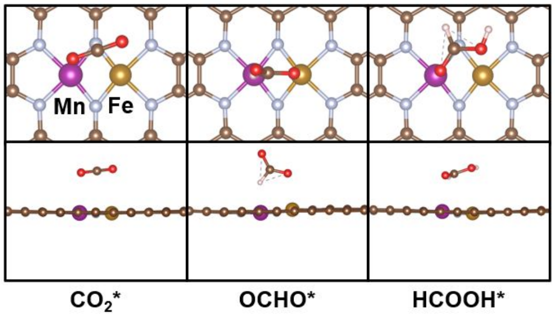

Figure S3 Optimized intermediates for CO_2 electroreduction on Mn/Fe DMSC site (brown: C; gray: N; red: O; white: H).

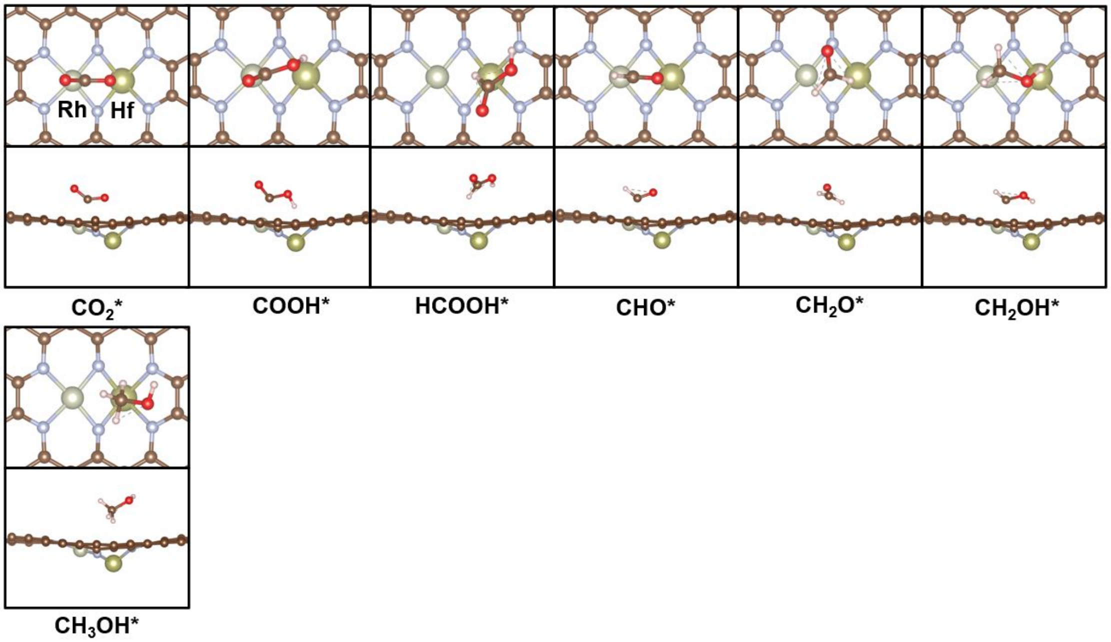

Figure S4 Optimized intermediates for CO_2 electroreduction to CH_3OH on Rh/Hf DMSC site (brown: C; gray: N; red: O; white: H).

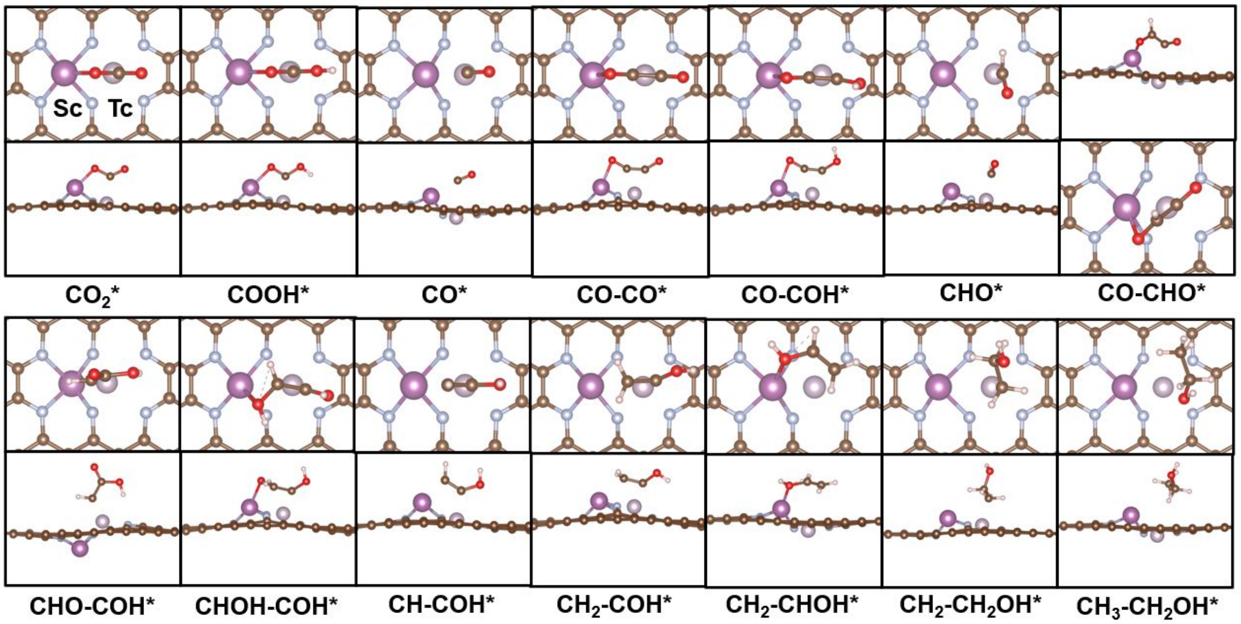

Figure S5 Optimized intermediates for CO_2 electroreduction to CH_3CH_2OH on Sc/Tc DMSC site (brown: C; gray: N; red: O; white: H).

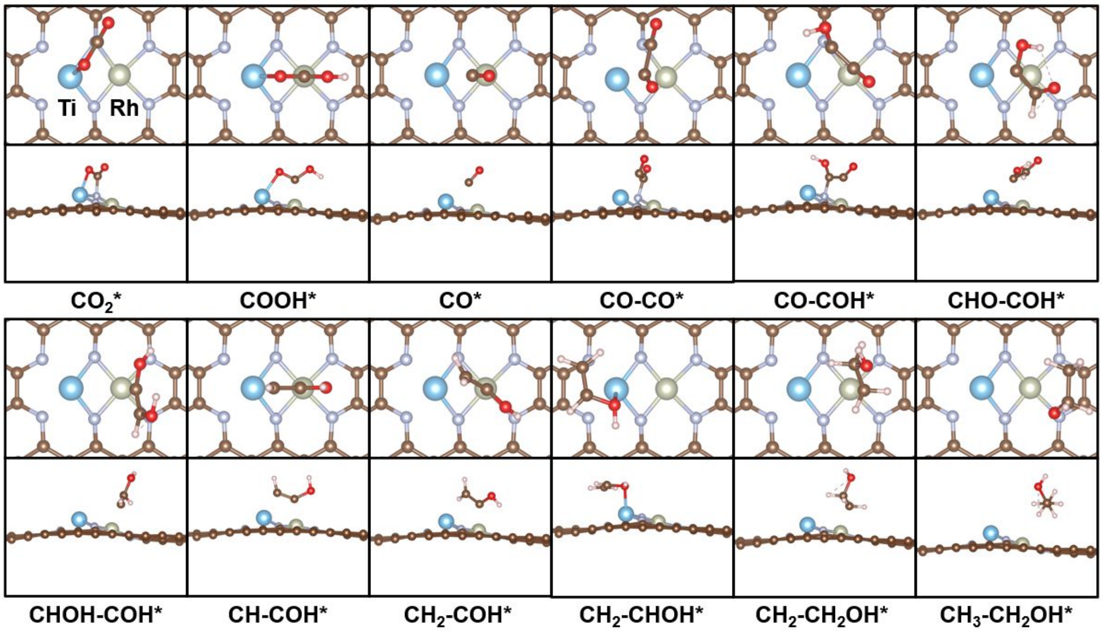

Figure S6 Optimized intermediates for CO_2 electroreduction to CH_3CH_2OH on Ti/Rh DMSC site (brown: C; gray: N; red: O; white: H).

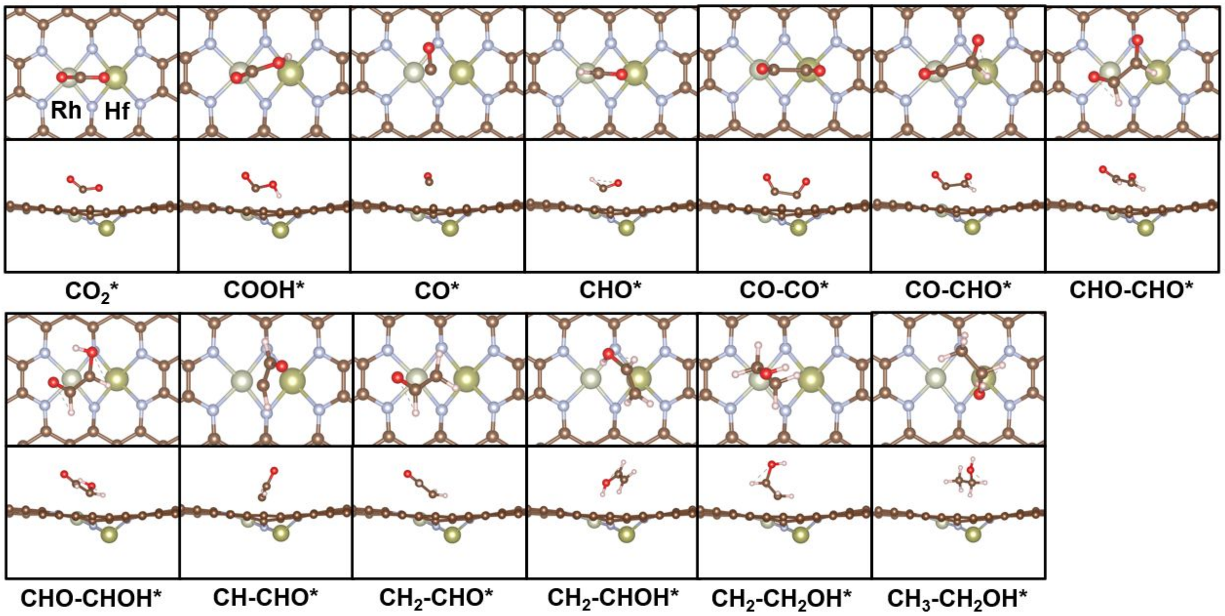

Figure S7 Optimized intermediates for CO_2 electroreduction to CH_3CH_2OH on Rh/Hf DMSC site (brown: C; gray: N; red: O; white: H).

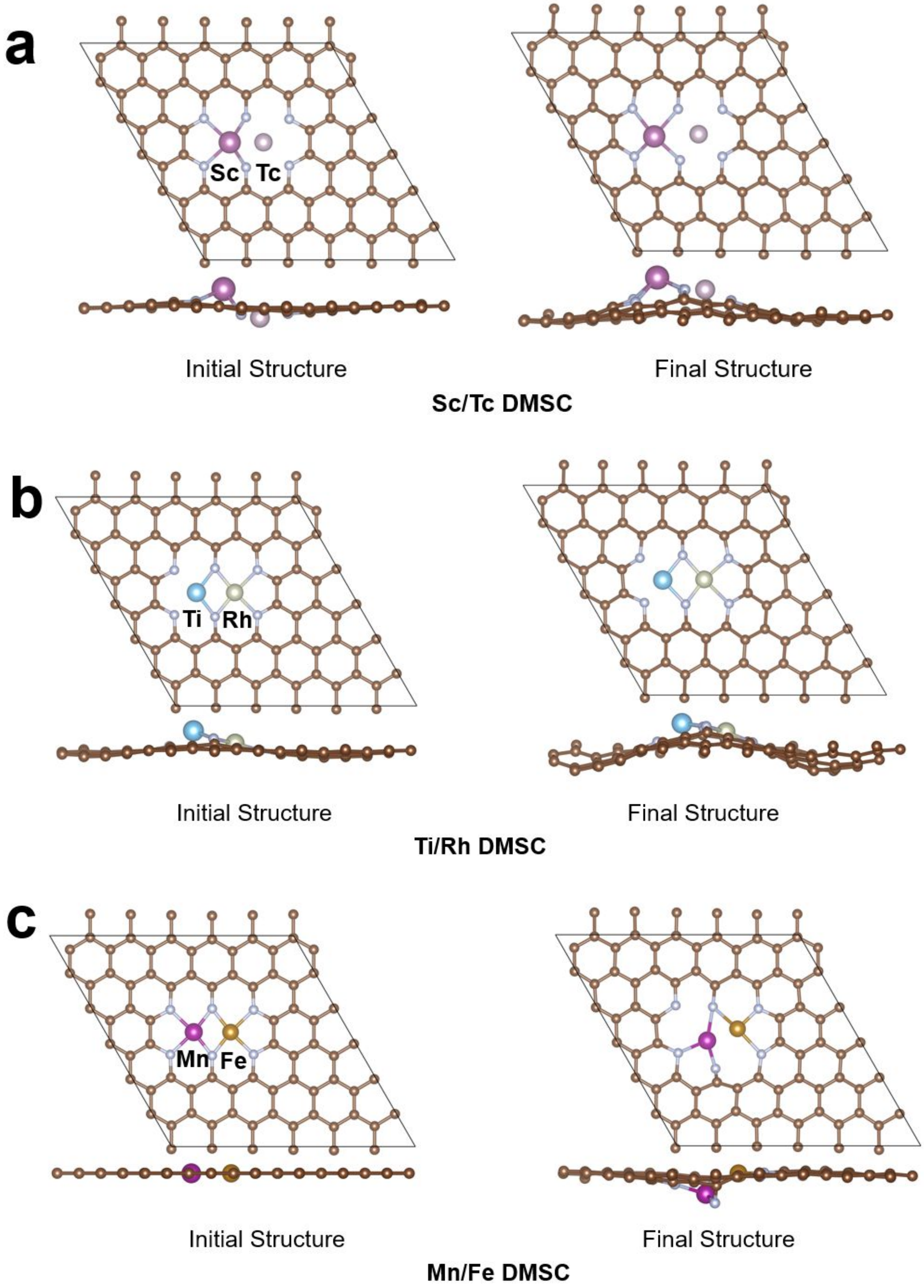
a
Sc
Tc
Initial Structure
Final Structure
Sc/Tc DMSC
b
Ti
Rh
Initial Structure
Final Structure
Ti/Rh DMSC
c
Mn
Fe
Initial Structure
Final Structure
Mn/Fe DMSC

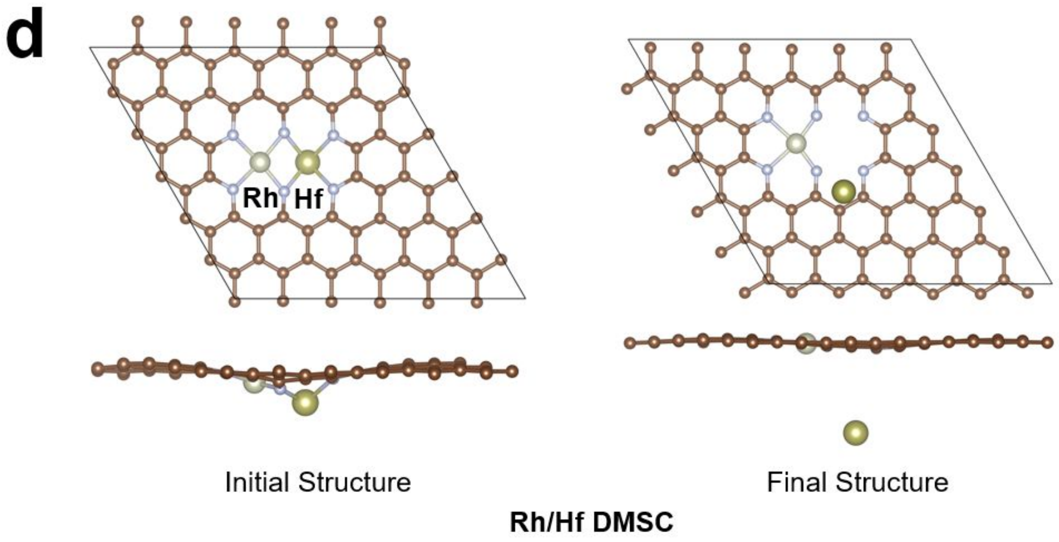

Figure S8 Structures of (a) Sc/Tc, (b) Ti/Rh, (c) Mn/Fe, (d) Rh/Hf DMSCs before (Initial structure) and after (Final structure) 300K AIMD simulations.

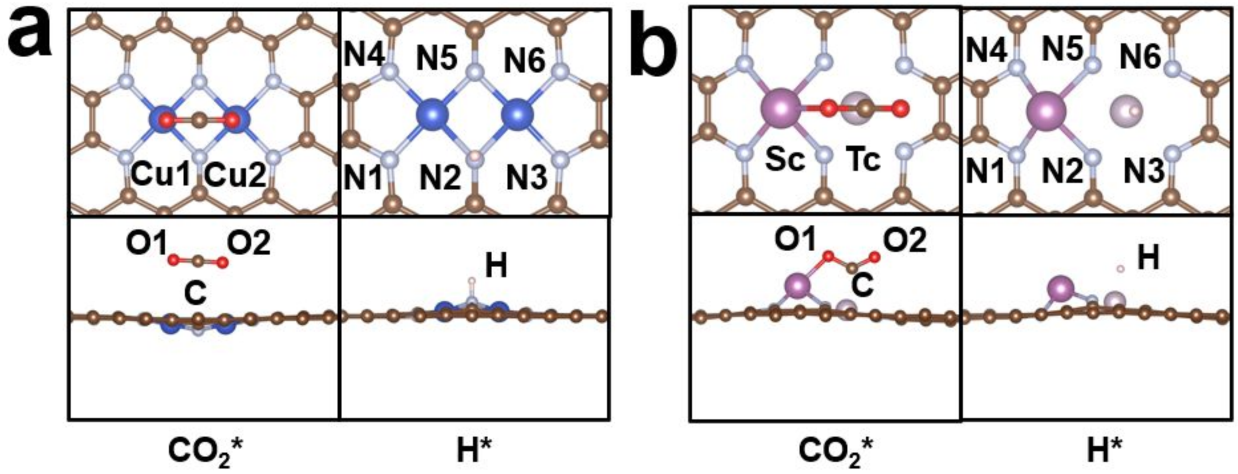

Figure S9 Position numbers of C, N, O, H and central metal atoms on (a) Cu/Cu DMSC and (b) Sc/Tc DMSC.

Supplementary Tables

Table S1 325 possible combinations of central metals and E_f and $E_{b\text{-}c}$ values.

ID	M1	M2	E_f	$E_{b\text{-}c}$	ID	M1	M2	E_f	$E_{b\text{-}c}$	ID	M1	M2	E_f	$E_{b\text{-}c}$
**1**	Sc	Sc	-14.98	-12.20	**110**	Mn	W	-17.48	-5.89	**219**	Mo	Pt	-13.81	-3.11
**2**	Sc	Ti	-15.73	-10.15	**111**	Mn	Re	-14.33	-5.65	**220**	Mo	Au	-8.28	-0.88
**3**	Sc	V	-15.30	-8.75	**112**	Mn	Os	-16.38	-5.31	**221**	Tc	Tc	-18.65	-5.73
**4**	Sc	Cr	-14.80	-9.42	**113**	Mn	Ir	-15.32	-6.05	**222**	Tc	Ru	-16.42	-4.40
**5**	Sc	Mn	-14.40	-9.30	**114**	Mn	Pt	-12.43	-5.36	**223**	Tc	Rh	-14.91	-5.42
**6**	Sc	Fe	-11.95	-9.86	**115**	Mn	Au	-7.02	-3.22	**224**	Tc	Pd	-11.27	-4.10
**7**	Sc	Co	-11.51	-9.02	**116**	Fe	Fe	-4.71	-3.18	**225**	Tc	Ag	-9.34	-3.05
**8**	Sc	Ni	-11.69	-9.35	**117**	Fe	Co	-4.97	-3.01	**226**	Tc	Cd	-8.38	-4.43
**9**	Sc	Cu	-9.47	-7.57	**118**	Fe	Ni	-6.12	-4.41	**227**	Tc	Hf	-19.01	-7.86
**10**	Sc	Zn	-7.22	-8.56	**119**	Fe	Cu	-5.84	-4.42	**228**	Tc	Ta	-20.15	-5.94
**11**	Sc	Mo	-16.56	-8.12	**120**	Fe	Zn	2.04	0.36	**229**	Tc	W	-18.01	-3.25
**12**	Sc	Tc	-16.47	-8.27	**121**	Fe	Mo	-13.93	-5.99	**230**	Tc	Re	-14.73	-2.90
**13**	Sc	Ru	-14.12	-7.32	**122**	Fe	Tc	-14.33	-7.05	**231**	Tc	Os	-16.87	-2.62
**14**	Sc	Rh	-12.77	-8.35	**123**	Fe	Ru	-11.26	-4.97	**232**	Tc	Ir	-15.88	-3.43
**15**	Sc	Pd	-10.33	-8.43	**124**	Fe	Rh	-5.64	-1.63	**233**	Tc	Pt	-12.97	-2.73
**16**	Sc	Ag	-7.24	-6.75	**125**	Fe	Pd	-0.44	0.99	**234**	Tc	Au	-7.47	-0.50
**17**	Sc	Cd	-5.69	-7.17	**126**	Fe	Ag	0.72	0.85	**235**	Ru	Ru	-14.46	-3.45
**18**	Sc	Hf	-17.48	-11.51	**127**	Fe	Cd	4.77	2.82	**236**	Ru	Rh	-12.40	-3.70
**19**	Sc	Ta	-17.35	-8.50	**128**	Fe	Hf	-11.02	-5.79	**237**	Ru	Pd	-8.95	-2.66
**20**	Sc	W	-17.30	-8.14	**129**	Fe	Ta	-11.81	-3.38	**238**	Ru	Ag	-4.98	-0.04
**21**	Sc	Re	-13.82	-7.24	**130**	Fe	W	-13.21	-4.79	**239**	Ru	Cd	-6.04	-3.17
**22**	Sc	Os	-15.88	-7.26	**131**	Fe	Re	-11.66	-5.86	**240**	Ru	Hf	-17.20	-7.18
**23**	Sc	Ir	-14.96	-7.94	**132**	Fe	Os	-12.27	-4.02	**241**	Ru	Ta	-18.06	-4.96
**24**	Sc	Pt	-13.00	-8.26	**133**	Fe	Ir	-7.39	-0.88	**242**	Ru	W	-17.62	-4.47
**25**	Sc	Au	-8.50	-7.15	**134**	Fe	Pt	-3.55	0.77	**243**	Ru	Re	-13.70	-3.15
**26**	Ti	Ti	-16.12	-7.77	**135**	Fe	Au	-0.51	0.41	**244**	Ru	Os	-15.84	-2.85
**27**	Ti	V	-16.19	-6.81	**136**	Co	Co	-7.60	-5.11	**245**	Ru	Ir	-14.51	-3.25

**28**	Ti	Cr	-15.85	-7.61	**137**	Co	Ni	-5.74	-3.54	**246**	Ru	Pt	-11.53	-2.43
**29**	Ti	Mn	-15.94	-8.14	**138**	Co	Cu	-5.62	-3.67	**247**	Ru	Au	-6.00	-0.18
**30**	Ti	Fe	-9.62	-4.70	**139**	Co	Zn	4.92	3.80	**248**	Rh	Rh	-10.31	-3.89
**31**	Ti	Co	-13.12	-7.60	**140**	Co	Mo	-12.28	-3.84	**249**	Rh	Pd	-6.71	-2.71
**32**	Ti	Ni	-12.85	-7.72	**141**	Co	Tc	-12.68	-4.79	**250**	Rh	Ag	-2.88	-0.16
**33**	Ti	Cu	-10.43	-5.77	**142**	Co	Ru	-10.41	-3.64	**251**	Rh	Cd	-3.17	-2.46
**34**	Ti	Zn	-8.18	-6.71	**143**	Co	Rh	-7.43	-2.92	**252**	Rh	Hf	-15.83	-8.13
**35**	Ti	Mo	-17.53	-6.18	**144**	Co	Pd	-0.01	1.99	**253**	Rh	Ta	-16.50	-5.86
**36**	Ti	Tc	-17.79	-7.02	**145**	Co	Ag	2.10	2.67	**254**	Rh	W	-15.69	-4.98
**37**	Ti	Ru	-15.74	-6.05	**146**	Co	Cd	2.44	1.01	**255**	Rh	Re	-12.01	-3.88
**38**	Ti	Rh	-14.22	-6.92	**147**	Co	Hf	-14.29	-8.42	**256**	Rh	Os	-13.77	-3.13
**39**	Ti	Pd	-11.41	-6.78	**148**	Co	Ta	-15.27	-6.35	**257**	Rh	Ir	-12.38	-3.40
**40**	Ti	Ag	-6.48	-2.91	**149**	Co	W	-12.89	-3.72	**258**	Rh	Pt	-9.28	-2.48
**41**	Ti	Cd	-6.77	-5.39	**150**	Co	Re	-11.25	-4.93	**259**	Rh	Au	-4.04	-0.54
**42**	Ti	Hf	-17.76	-8.97	**151**	Co	Os	-11.55	-2.80	**260**	Pd	Pd	-3.63	-2.18
**43**	Ti	Ta	-18.12	-6.24	**152**	Co	Ir	-7.79	-0.70	**261**	Pd	Ag	-0.59	-0.36
**44**	Ti	W	-18.11	-6.03	**153**	Co	Pt	-3.12	1.70	**262**	Pd	Cd	-0.33	-2.44
**45**	Ti	Re	-15.12	-5.87	**154**	Co	Au	1.34	2.70	**263**	Pd	Hf	-13.20	-8.10
**46**	Ti	Os	-16.20	-4.33	**155**	Ni	Ni	-1.88	-0.10	**264**	Pd	Ta	-13.26	-5.30
**47**	Ti	Ir	-15.47	-5.36	**156**	Ni	Cu	-5.33	-3.80	**265**	Pd	W	-11.94	-3.38
**48**	Ti	Pt	-13.07	-5.27	**157**	Ni	Zn	-0.40	-1.85	**266**	Pd	Re	-8.58	-2.94
**49**	Ti	Au	-7.67	-3.25	**158**	Ni	Mo	-10.53	-2.31	**267**	Pd	Os	-10.20	-2.00
**50**	V	V	-16.40	-6.29	**159**	Ni	Tc	-10.74	-3.10	**268**	Pd	Ir	-8.70	-2.18
**51**	V	Cr	-15.94	-6.66	**160**	Ni	Ru	-10.62	-3.99	**269**	Pd	Pt	-6.11	-1.89
**52**	V	Mn	-15.90	-7.10	**161**	Ni	Rh	-6.17	-1.83	**270**	Pd	Au	-1.80	-0.82
**53**	V	Fe	-11.77	-5.87	**162**	Ni	Pd	-3.63	-2.01	**271**	Ag	Ag	2.72	1.65
**54**	V	Co	-12.41	-6.01	**163**	Ni	Ag	1.35	1.77	**272**	Ag	Cd	2.51	-0.94
**55**	V	Ni	-12.50	-6.32	**164**	Ni	Cd	3.73	1.88	**273**	Ag	Hf	-9.82	-6.17
**56**	V	Cu	-9.98	-4.57	**165**	Ni	Hf	-10.37	-4.80	**274**	Ag	Ta	-10.31	-3.11
**57**	V	Zn	-7.21	-4.49	**166**	Ni	Ta	-9.29	-0.85	**275**	Ag	W	-7.98	-0.79
**58**	V	Mo	-17.77	-5.61	**167**	Ni	W	-9.70	-0.79	**276**	Ag	Re	-4.62	-0.36
**59**	V	Tc	-17.52	-5.67	**168**	Ni	Re	-10.31	-4.20	**277**	Ag	Os	-6.20	0.60

**60**	V	Ru	-15.53	-4.73	**169**	Ni	Os	-9.71	-1.13	**278**	Ag	Ir	-4.76	0.49
**61**	V	Rh	-13.91	-5.55	**170**	Ni	Ir	-8.16	-1.27	**279**	Ag	Pt	-3.04	-0.03
**62**	V	Pd	-10.59	-4.73	**171**	Ni	Pt	-6.15	-1.73	**280**	Ag	Au	1.47	1.21
**63**	V	Ag	-7.67	-3.18	**172**	Ni	Au	2.36	3.57	**281**	Cd	Cd	4.19	-1.25
**64**	V	Cd	-6.44	-4.22	**173**	Cu	Cu	-3.81	-1.53	**282**	Cd	Hf	-8.58	-6.81
**65**	V	Hf	-17.51	-7.65	**174**	Cu	Zn	-1.11	-2.82	**283**	Cd	Ta	-8.81	-4.07
**66**	V	Ta	-18.10	-5.29	**175**	Cu	Mo	-10.98	-3.60	**284**	Cd	W	-9.42	-4.28
**67**	V	W	-18.27	-5.36	**176**	Cu	Tc	-10.88	-3.87	**285**	Cd	Re	-2.62	-0.94
**68**	V	Re	-14.86	-4.52	**177**	Cu	Ru	-8.16	-1.87	**286**	Cd	Os	-7.55	-2.70
**69**	V	Os	-16.90	-4.11	**178**	Cu	Rh	-5.96	-1.95	**287**	Cd	Ir	-5.46	-2.16
**70**	V	Ir	-16.10	-5.13	**179**	Cu	Pd	-3.33	-1.95	**288**	Cd	Pt	-3.02	-2.26
**71**	V	Pt	-13.26	-4.56	**180**	Cu	Ag	-1.06	-1.55	**289**	Cd	Au	1.30	-1.36
**72**	V	Au	-7.84	-2.45	**181**	Cu	Cd	0.23	-1.83	**290**	Hf	Hf	-19.46	-10.33
**73**	Cr	Cr	-15.96	-8.06	**182**	Cu	Hf	-12.24	-7.20	**291**	Hf	Ta	-19.24	-7.04
**74**	Cr	Mn	-15.77	-8.28	**183**	Cu	Ta	-12.49	-4.47	**292**	Hf	W	-19.54	-7.07
**75**	Cr	Fe	-13.03	-8.39	**184**	Cu	W	-11.67	-3.56	**293**	Hf	Re	-16.31	-6.69
**76**	Cr	Co	-12.86	-7.69	**185**	Cu	Re	-8.30	-3.06	**294**	Hf	Os	-18.62	-6.62
**77**	Cr	Ni	-10.16	-5.14	**186**	Cu	Os	-10.24	-2.66	**295**	Hf	Ir	-17.99	-7.66
**78**	Cr	Cu	-9.15	-4.60	**187**	Cu	Ir	-8.24	-1.91	**296**	Hf	Pt	-15.84	-7.88
**79**	Cr	Zn	-6.79	-5.34	**188**	Cu	Pt	-5.87	-1.69	**297**	Hf	Au	-10.87	-6.40
**80**	Cr	Mo	-17.59	-6.69	**189**	Cu	Au	-2.17	-1.88	**298**	Ta	Ta	-19.90	-4.41
**81**	Cr	Tc	-17.38	-6.79	**190**	Zn	Zn	1.90	-2.81	**299**	Ta	W	-19.90	-4.22
**82**	Cr	Ru	-15.29	-5.82	**191**	Zn	Mo	-7.94	-3.21	**300**	Ta	Re	-15.54	-2.81
**83**	Cr	Rh	-17.61	-10.54	**192**	Zn	Tc	-8.99	-4.97	**301**	Ta	Os	-19.75	-4.90
**84**	Cr	Pd	-4.83	-0.48	**193**	Zn	Ru	-5.82	-2.62	**302**	Ta	Ir	-18.62	-5.39
**85**	Cr	Ag	-6.37	-3.26	**194**	Zn	Rh	-3.52	-2.58	**303**	Ta	Pt	-15.89	-5.12
**86**	Cr	Cd	-6.31	-5.25	**195**	Zn	Pd	-0.91	-2.62	**304**	Ta	Au	-10.41	-3.12
**87**	Cr	Hf	-17.19	-8.48	**196**	Zn	Ag	1.81	-1.07	**305**	W	W	-19.45	-3.48
**88**	Cr	Ta	-18.21	-6.65	**197**	Zn	Cd	2.37	-2.96	**306**	W	Re	-16.93	-4.08
**89**	Cr	W	-18.13	-6.50	**198**	Zn	Hf	-3.90	-1.61	**307**	W	Os	-18.01	-2.50
**90**	Cr	Re	-14.59	-5.48	**199**	Zn	Ta	-10.07	-5.36	**308**	W	Ir	-17.19	-3.51
**91**	Cr	Os	-16.65	-5.16	**200**	Zn	W	-9.99	-4.83	**309**	W	Pt	-14.56	-3.16

**92**	Cr	Ir	-15.59	-5.92	**201**	Zn	Re	-6.49	-4.09	**310**	W	Au	-8.99	-0.92
**93**	Cr	Pt	-12.85	-5.41	**202**	Zn	Os	-8.19	-3.30	**311**	Re	Re	-13.38	-3.26
**94**	Cr	Au	-7.43	-3.32	**203**	Zn	Ir	-6.19	-2.89	**312**	Re	Os	-14.89	-2.39
**95**	Mn	Mn	-15.58	-8.51	**204**	Zn	Pt	-3.46	-2.36	**313**	Re	Ir	-14.08	-3.41
**96**	Mn	Fe	-12.97	-8.62	**205**	Zn	Au	0.70	-1.43	**314**	Re	Pt	-11.12	-2.72
**97**	Mn	Co	-12.88	-8.06	**206**	Mo	Mo	-19.52	-5.44	**315**	Re	Au	-5.49	-0.41
**98**	Mn	Ni	-9.63	-5.05	**207**	Mo	Tc	-19.39	-5.75	**316**	Os	Os	-17.17	-2.19
**99**	Mn	Cu	-8.78	-4.61	**208**	Mo	Ru	-17.15	-4.75	**317**	Os	Ir	-15.83	-2.64
**100**	Mn	Zn	-6.17	-5.06	**209**	Mo	Rh	-14.43	-4.10	**318**	Os	Pt	-12.74	-1.73
**101**	Mn	Mo	-16.93	-6.11	**210**	Mo	Pd	-11.17	-3.31	**319**	Os	Au	-7.07	0.63
**102**	Mn	Tc	-17.11	-6.91	**211**	Mo	Ag	-7.21	-0.70	**320**	Ir	Ir	-14.40	-2.89
**103**	Mn	Ru	-15.01	-5.94	**212**	Mo	Cd	-8.57	-4.17	**321**	Ir	Pt	-11.23	-1.91
**104**	Mn	Rh	-13.14	-6.45	**213**	Mo	Hf	-18.89	-7.13	**322**	Ir	Au	-5.85	0.12
**105**	Mn	Pd	-9.77	-5.55	**214**	Mo	Ta	-19.76	-5.07	**323**	Pt	Pt	-8.54	-1.62
**106**	Mn	Ag	-6.02	-3.56	**215**	Mo	W	-20.04	-5.17	**324**	Pt	Au	-4.21	-0.47
**107**	Mn	Cd	-5.78	-4.99	**216**	Mo	Re	-15.69	-3.40	**325**	Au	Au	0.28	0.75
**108**	Mn	Hf	-17.18	-9.05	**217**	Mo	Os	-17.70	-2.95					
**109**	Mn	Ta	-18.22	-7.00	**218**	Mo	Ir	-16.58	-3.64					

Table S2 List of 295 stable DMACs.

**ID**	**M1**	**M2**	**ID**	**M1**	**M2**	**ID**	**M1**	**M2**	**ID**	**M1**	**M2**	**ID**	**M1**	**M2**	**ID**	**M1**	**M2**
**1**	Sc	Sc	**51**	V	Cr	**101**	Mn	Mo	**165**	Ni	Hf	**221**	Tc	Tc	**273**	Ag	Hf
**2**	Sc	Ti	**52**	V	Mn	**102**	Mn	Tc	**166**	Ni	Ta	**222**	Tc	Ru	**274**	Ag	Ta
**3**	Sc	V	**53**	V	Fe	**103**	Mn	Ru	**167**	Ni	W	**223**	Tc	Rh	**275**	Ag	W
**4**	Sc	Cr	**54**	V	Co	**104**	Mn	Rh	**168**	Ni	Re	**224**	Tc	Pd	**276**	Ag	Re
**5**	Sc	Mn	**55**	V	Ni	**105**	Mn	Pd	**169**	Ni	Os	**225**	Tc	Ag	**279**	Ag	Pt
**6**	Sc	Fe	**56**	V	Cu	**106**	Mn	Ag	**170**	Ni	Ir	**226**	Tc	Cd	**282**	Cd	Hf
**7**	Sc	Co	**57**	V	Zn	**107**	Mn	Cd	**171**	Ni	Pt	**227**	Tc	Hf	**283**	Cd	Ta
**8**	Sc	Ni	**58**	V	Mo	**108**	Mn	Hf	**173**	Cu	Cu	**228**	Tc	Ta	**284**	Cd	W
**9**	Sc	Cu	**59**	V	Tc	**109**	Mn	Ta	**174**	Cu	Zn	**229**	Tc	W	**285**	Cd	Re
**10**	Sc	Zn	**60**	V	Ru	**110**	Mn	W	**175**	Cu	Mo	**230**	Tc	Re	**286**	Cd	Os

**11**	Sc	Mo	**61**	V	Rh	**111**	Mn	Re	**176**	Cu	Tc	**231**	Tc	Os	**287**	Cd	Ir
**12**	Sc	Tc	**62**	V	Pd	**112**	Mn	Os	**177**	Cu	Ru	**232**	Tc	Ir	**288**	Cd	Pt
**13**	Sc	Ru	**63**	V	Ag	**113**	Mn	Ir	**178**	Cu	Rh	**233**	Tc	Pt	**290**	Hf	Hf
**14**	Sc	Rh	**64**	V	Cd	**114**	Mn	Pt	**179**	Cu	Pd	**234**	Tc	Au	**291**	Hf	Ta
**15**	Sc	Pd	**65**	V	Hf	**115**	Mn	Au	**180**	Cu	Ag	**235**	Ru	Ru	**292**	Hf	W
**16**	Sc	Ag	**66**	V	Ta	**116**	Fe	Fe	**182**	Cu	Hf	**236**	Ru	Rh	**293**	Hf	Re
**17**	Sc	Cd	**67**	V	W	**117**	Fe	Co	**183**	Cu	Ta	**237**	Ru	Pd	**294**	Hf	Os
**18**	Sc	Hf	**68**	V	Re	**118**	Fe	Ni	**184**	Cu	W	**238**	Ru	Ag	**295**	Hf	Ir
**19**	Sc	Ta	**69**	V	Os	**119**	Fe	Cu	**185**	Cu	Re	**239**	Ru	Cd	**296**	Hf	Pt
**20**	Sc	W	**70**	V	Ir	**121**	Fe	Mo	**186**	Cu	Os	**240**	Ru	Hf	**297**	Hf	Au
**21**	Sc	Re	**71**	V	Pt	**122**	Fe	Tc	**187**	Cu	Ir	**241**	Ru	Ta	**298**	Ta	Ta
**22**	Sc	Os	**72**	V	Au	**123**	Fe	Ru	**188**	Cu	Pt	**242**	Ru	W	**299**	Ta	W
**23**	Sc	Ir	**73**	Cr	Cr	**124**	Fe	Rh	**189**	Cu	Au	**243**	Ru	Re	**300**	Ta	Re
**24**	Sc	Pt	**74**	Cr	Mn	**128**	Fe	Hf	**191**	Zn	Mo	**244**	Ru	Os	**301**	Ta	Os
**25**	Sc	Au	**75**	Cr	Fe	**129**	Fe	Ta	**192**	Zn	Tc	**245**	Ru	Ir	**302**	Ta	Ir
**26**	Ti	Ti	**76**	Cr	Co	**130**	Fe	W	**193**	Zn	Ru	**246**	Ru	Pt	**303**	Ta	Pt
**27**	Ti	V	**77**	Cr	Ni	**131**	Fe	Re	**194**	Zn	Rh	**247**	Ru	Au	**304**	Ta	Au
**28**	Ti	Cr	**78**	Cr	Cu	**132**	Fe	Os	**195**	Zn	Pd	**248**	Rh	Rh	**305**	W	W
**29**	Ti	Mn	**79**	Cr	Zn	**133**	Fe	Ir	**198**	Zn	Hf	**249**	Rh	Pd	**306**	W	Re
**30**	Ti	Fe	**80**	Cr	Mo	**136**	Co	Co	**199**	Zn	Ta	**250**	Rh	Ag	**307**	W	Os
**31**	Ti	Co	**81**	Cr	Tc	**137**	Co	Ni	**200**	Zn	W	**251**	Rh	Cd	**308**	W	Ir
**32**	Ti	Ni	**82**	Cr	Ru	**138**	Co	Cu	**201**	Zn	Re	**252**	Rh	Hf	**309**	W	Pt
**33**	Ti	Cu	**83**	Cr	Rh	**140**	Co	Mo	**202**	Zn	Os	**253**	Rh	Ta	**310**	W	Au
**34**	Ti	Zn	**84**	Cr	Pd	**141**	Co	Tc	**203**	Zn	Ir	**254**	Rh	W	**311**	Re	Re
**35**	Ti	Mo	**85**	Cr	Ag	**142**	Co	Ru	**204**	Zn	Pt	**255**	Rh	Re	**312**	Re	Os
**36**	Ti	Tc	**86**	Cr	Cd	**143**	Co	Rh	**206**	Mo	Mo	**256**	Rh	Os	**313**	Re	Ir
**37**	Ti	Ru	**87**	Cr	Hf	**147**	Co	Hf	**207**	Mo	Tc	**257**	Rh	Ir	**314**	Re	Pt
**38**	Ti	Rh	**88**	Cr	Ta	**148**	Co	Ta	**208**	Mo	Ru	**258**	Rh	Pt	**315**	Re	Au
**39**	Ti	Pd	**89**	Cr	W	**149**	Co	W	**209**	Mo	Rh	**259**	Rh	Au	**316**	Os	Os
**40**	Ti	Ag	**90**	Cr	Re	**150**	Co	Re	**210**	Mo	Pd	**260**	Pd	Pd	**317**	Os	Ir
**41**	Ti	Cd	**91**	Cr	Os	**151**	Co	Os	**211**	Mo	Ag	**261**	Pd	Ag	**318**	Os	Pt
**42**	Ti	Hf	**92**	Cr	Ir	**152**	Co	Ir	**212**	Mo	Cd	**262**	Pd	Cd	**320**	Ir	Ir

**43**	Ti	Ta	**93**	Cr	Pt	**155**	Ni	Ni	**213**	Mo	Hf	**263**	Pd	Hf	**321**	Ir	Pt
**44**	Ti	W	**94**	Cr	Au	**156**	Ni	Cu	**214**	Mo	Ta	**264**	Pd	Ta	**323**	Pt	Pt
**45**	Ti	Re	**95**	Mn	Mn	**157**	Ni	Zn	**215**	Mo	W	**265**	Pd	W	**324**	Pt	Au
**46**	Ti	Os	**96**	Mn	Fe	**158**	Ni	Mo	**216**	Mo	Re	**266**	Pd	Re			
**47**	Ti	Ir	**97**	Mn	Co	**159**	Ni	Tc	**217**	Mo	Os	**267**	Pd	Os			
**48**	Ti	Pt	**98**	Mn	Ni	**160**	Ni	Ru	**218**	Mo	Ir	**268**	Pd	Ir			
**49**	Ti	Au	**99**	Mn	Cu	**161**	Ni	Rh	**219**	Mo	Pt	**269**	Pd	Pt			
**50**	V	V	**100**	Mn	Zn	**162**	Ni	Pd	**220**	Mo	Au	**270**	Pd	Au			

Table S3 E_{ads}(CO) values of 295 stable DMACs.

**ID**	**M1**	**M2**	**E_{ads}(CO)**	**ID**	**M1**	**M2**	**E_{ads}(CO)**	**ID**	**M1**	**M2**	**E_{ads}(CO)**	**ID**	**M1**	**M2**	**E_{ads}(CO)**
**1**	Sc	Sc	0.52	**75**	Cr	Fe	-0.38	**161**	Ni	Rh	-2.38	**243**	Ru	Re	-1.52
**2**	Sc	Ti	0.53	**76**	Cr	Co	0.06	**162**	Ni	Pd	-1.80	**244**	Ru	Os	-1.00
**3**	Sc	V	-1.11	**77**	Cr	Ni	-1.03	**165**	Ni	Hf	-3.72	**245**	Ru	Ir	-0.60
**4**	Sc	Cr	-1.20	**78**	Cr	Cu	-1.05	**166**	Ni	Ta	-3.41	**246**	Ru	Pt	-0.86
**5**	Sc	Mn	-0.90	**79**	Cr	Zn	-1.54	**167**	Ni	W	-1.41	**247**	Ru	Au	-1.83
**6**	Sc	Fe	-0.37	**80**	Cr	Mo	-0.90	**168**	Ni	Re	-1.03	**248**	Rh	Rh	0.51
**7**	Sc	Co	-0.36	**81**	Cr	Tc	-0.52	**169**	Ni	Os	-3.02	**249**	Rh	Pd	-0.31
**8**	Sc	Ni	0.36	**82**	Cr	Ru	-0.79	**170**	Ni	Ir	-2.41	**250**	Rh	Ag	-1.61
**9**	Sc	Cu	0.42	**83**	Cr	Rh	3.41	**171**	Ni	Pt	-1.88	**251**	Rh	Cd	0.04
**10**	Sc	Zn	0.45	**84**	Cr	Pd	-6.64	**173**	Cu	Cu	0.29	**252**	Rh	Hf	0.28
**11**	Sc	Mo	-0.95	**85**	Cr	Ag	-1.67	**174**	Cu	Zn	0.28	**253**	Rh	Ta	-0.22
**12**	Sc	Tc	0.15	**86**	Cr	Cd	-0.84	**175**	Cu	Mo	-0.82	**254**	Rh	W	-0.64
**13**	Sc	Ru	-1.69	**87**	Cr	Hf	-1.10	**176**	Cu	Tc	-0.46	**255**	Rh	Re	-1.25
**14**	Sc	Rh	-0.08	**88**	Cr	Ta	-1.04	**177**	Cu	Ru	-0.93	**256**	Rh	Os	-0.97
**15**	Sc	Pd	0.44	**89**	Cr	W	-0.74	**178**	Cu	Rh	-0.79	**257**	Rh	Ir	0.30
**16**	Sc	Ag	0.46	**90**	Cr	Re	-1.06	**179**	Cu	Pd	-0.01	**258**	Rh	Pt	-0.27
**17**	Sc	Cd	0.40	**91**	Cr	Os	-1.21	**180**	Cu	Ag	0.22	**259**	Rh	Au	-1.10
**18**	Sc	Hf	0.52	**92**	Cr	Ir	-1.13	**182**	Cu	Hf	0.36	**260**	Pd	Pd	-0.14
**19**	Sc	Ta	-0.16	**93**	Cr	Pt	-0.93	**183**	Cu	Ta	-0.41	**261**	Pd	Ag	-1.00
**20**	Sc	W	0.30	**94**	Cr	Au	-1.66	**184**	Cu	W	-0.43	**262**	Pd	Cd	0.42

**21**	Sc	Re	-0.98	**95**	Mn	Mn	-0.56	**185**	Cu	Re	-0.47	**263**	Pd	Hf	0.43
**22**	Sc	Os	-1.44	**96**	Mn	Fe	-0.08	**186**	Cu	Os	-0.46	**264**	Pd	Ta	0.40
**23**	Sc	Ir	-0.32	**97**	Mn	Co	0.42	**187**	Cu	Ir	-0.62	**265**	Pd	W	-1.99
**24**	Sc	Pt	0.41	**98**	Mn	Ni	0.03	**188**	Cu	Pt	-0.14	**266**	Pd	Re	-1.38
**25**	Sc	Au	0.52	**99**	Mn	Cu	-0.68	**189**	Cu	Au	0.42	**267**	Pd	Os	-1.06
**26**	Ti	Ti	0.24	**100**	Mn	Zn	-0.87	**191**	Zn	Mo	-2.06	**268**	Pd	Ir	-0.74
**27**	Ti	V	-1.12	**101**	Mn	Mo	-1.18	**192**	Zn	Tc	-0.04	**269**	Pd	Pt	-0.26
**28**	Ti	Cr	-1.38	**102**	Mn	Tc	-0.65	**193**	Zn	Ru	-1.16	**270**	Pd	Au	-0.38
**29**	Ti	Mn	-0.98	**103**	Mn	Ru	-0.72	**194**	Zn	Rh	-1.13	**273**	Ag	Hf	0.40
**30**	Ti	Fe	-4.15	**104**	Mn	Rh	-0.49	**195**	Zn	Pd	-0.45	**274**	Ag	Ta	-0.54
**31**	Ti	Co	0.23	**105**	Mn	Pd	-0.57	**198**	Zn	Hf	-5.67	**275**	Ag	W	-3.14
**32**	Ti	Ni	0.47	**106**	Mn	Ag	-1.04	**199**	Zn	Ta	0.29	**276**	Ag	Re	-2.71
**33**	Ti	Cu	0.16	**107**	Mn	Cd	-0.23	**200**	Zn	W	-0.86	**279**	Ag	Pt	-0.90
**34**	Ti	Zn	0.12	**108**	Mn	Hf	-0.74	**201**	Zn	Re	-0.40	**282**	Cd	Hf	0.23
**35**	Ti	Mo	-0.33	**109**	Mn	Ta	-0.66	**202**	Zn	Os	-0.24	**283**	Cd	Ta	0.20
**36**	Ti	Tc	-0.88	**110**	Mn	W	-1.42	**203**	Zn	Ir	-0.56	**284**	Cd	W	-0.34
**37**	Ti	Ru	-1.35	**111**	Mn	Re	-1.20	**204**	Zn	Pt	-0.85	**285**	Cd	Re	-3.30
**38**	Ti	Rh	0.08	**112**	Mn	Os	-1.00	**206**	Mo	Mo	0.30	**286**	Cd	Os	0.21
**39**	Ti	Pd	0.12	**113**	Mn	Ir	-0.44	**207**	Mo	Tc	-0.24	**287**	Cd	Ir	-0.23
**40**	Ti	Ag	-1.33	**114**	Mn	Pt	-0.55	**208**	Mo	Ru	-0.92	**288**	Cd	Pt	-0.22
**41**	Ti	Cd	0.17	**115**	Mn	Au	-0.70	**209**	Mo	Rh	-1.78	**290**	Hf	Hf	0.31
**42**	Ti	Hf	-0.23	**116**	Fe	Fe	-4.69	**210**	Mo	Pd	-1.91	**291**	Hf	Ta	-0.44
**43**	Ti	Ta	-0.28	**117**	Fe	Co	-3.97	**211**	Mo	Ag	-3.29	**292**	Hf	W	-1.31
**44**	Ti	W	-0.28	**118**	Fe	Ni	-2.18	**212**	Mo	Cd	0.22	**293**	Hf	Re	-1.48
**45**	Ti	Re	-1.24	**119**	Fe	Cu	-0.21	**213**	Mo	Hf	-1.05	**294**	Hf	Os	-1.67
**46**	Ti	Os	-2.48	**121**	Fe	Mo	-0.40	**214**	Mo	Ta	-0.88	**295**	Hf	Ir	0.18
**47**	Ti	Ir	-0.78	**122**	Fe	Tc	0.45	**215**	Mo	W	0.34	**296**	Hf	Pt	0.43
**48**	Ti	Pt	-0.84	**123**	Fe	Ru	-0.50	**216**	Mo	Re	-1.72	**297**	Hf	Au	0.49
**49**	Ti	Au	-1.32	**124**	Fe	Rh	-4.08	**217**	Mo	Os	-1.80	**298**	Ta	Ta	-0.70
**50**	V	V	-0.71	**128**	Fe	Hf	-4.09	**218**	Mo	Ir	-1.83	**299**	Ta	W	-0.50
**51**	V	Cr	-1.04	**129**	Fe	Ta	-3.87	**219**	Mo	Pt	-1.89	**300**	Ta	Re	-3.22
**52**	V	Mn	-0.97	**130**	Fe	W	-0.96	**220**	Mo	Au	-2.30	**301**	Ta	Os	0.38

**53**	V	Fe	-1.68	**131**	Fe	Re	-0.95	**221**	Tc	Tc	-1.04	**302**	Ta	Ir	0.33
**54**	V	Co	-0.84	**132**	Fe	Os	-0.89	**222**	Tc	Ru	-1.20	**303**	Ta	Pt	0.36
**55**	V	Ni	-0.71	**133**	Fe	Ir	-4.49	**223**	Tc	Rh	-0.18	**304**	Ta	Au	0.42
**56**	V	Cu	-1.01	**136**	Co	Co	-0.69	**224**	Tc	Pd	-0.84	**305**	W	W	-1.87
**57**	V	Zn	-1.53	**137**	Co	Ni	-2.08	**225**	Tc	Ag	-0.25	**306**	W	Re	-0.76
**58**	V	Mo	-0.77	**138**	Co	Cu	-0.07	**226**	Tc	Cd	0.37	**307**	W	Os	-2.30
**59**	V	Tc	-1.04	**140**	Co	Mo	1.56	**227**	Tc	Hf	-0.72	**308**	W	Ir	-2.06
**60**	V	Ru	-1.07	**141**	Co	Tc	0.23	**228**	Tc	Ta	-0.53	**309**	W	Pt	-1.99
**61**	V	Rh	-1.07	**142**	Co	Ru	-0.42	**229**	Tc	W	-2.74	**310**	W	Au	-2.44
**62**	V	Pd	-1.32	**143**	Co	Rh	-1.51	**230**	Tc	Re	-2.73	**311**	Re	Re	-0.88
**63**	V	Ag	-1.04	**147**	Co	Hf	0.60	**231**	Tc	Os	-2.37	**312**	Re	Os	-1.71
**64**	V	Cd	-0.95	**148**	Co	Ta	0.17	**232**	Tc	Ir	-1.72	**313**	Re	Ir	-1.30
**65**	V	Hf	-1.05	**149**	Co	W	1.74	**233**	Tc	Pt	-1.78	**314**	Re	Pt	-1.45
**66**	V	Ta	-1.09	**150**	Co	Re	-0.98	**234**	Tc	Au	-1.95	**315**	Re	Au	-1.69
**67**	V	W	-0.79	**151**	Co	Os	-0.06	**235**	Ru	Ru	-0.94	**316**	Os	Os	-0.99
**68**	V	Re	-1.43	**152**	Co	Ir	-3.41	**236**	Ru	Rh	-0.67	**317**	Os	Ir	-0.96
**69**	V	Os	-1.29	**155**	Ni	Ni	-5.33	**237**	Ru	Pd	-0.84	**318**	Os	Pt	-1.09
**70**	V	Ir	-1.09	**156**	Ni	Cu	0.35	**238**	Ru	Ag	-2.45	**320**	Ir	Ir	1.08
**71**	V	Pt	-1.36	**157**	Ni	Zn	-2.69	**239**	Ru	Cd	0.29	**321**	Ir	Pt	-0.72
**72**	V	Au	-1.69	**158**	Ni	Mo	-3.85	**240**	Ru	Hf	-1.31	**323**	Pt	Pt	-0.29
**73**	Cr	Cr	-0.74	**159**	Ni	Tc	-1.91	**241**	Ru	Ta	-0.77	**324**	Pt	Au	-0.36
**74**	Cr	Mn	-0.84	**160**	Ni	Ru	-0.55	**242**	Ru	W	0.09				

Table S4 List of 46 DMSCs with moderate E_{ads}(CO).

ID	M1	M2	E_{ads}(CO)	ID	M1	M2	E_{ads}(CO)	ID	M1	M2	E_{ads}(CO)	ID	M1	M2	E_{ads}(CO)
**12**	Sc	Tc	0.15	**44**	Ti	W	-0.28	**180**	Cu	Ag	0.22	**258**	Rh	Pt	-0.27
**14**	Sc	Rh	-0.08	**76**	Cr	Co	0.06	**188**	Cu	Pt	-0.14	**260**	Pd	Pd	-0.14
**19**	Sc	Ta	-0.16	**96**	Mn	Fe	-0.08	**192**	Zn	Tc	-0.04	**269**	Pd	Pt	-0.26
**26**	Ti	Ti	0.24	**98**	Mn	Ni	0.03	**202**	Zn	Os	-0.24	**282**	Cd	Hf	0.23
**31**	Ti	Co	0.23	**107**	Mn	Cd	-0.23	**207**	Mo	Tc	-0.24	**283**	Cd	Ta	0.20
**33**	Ti	Cu	0.16	**119**	Fe	Cu	-0.21	**212**	Mo	Cd	0.22	**286**	Cd	Os	0.21

**34**	Ti	Zn	0.12	**138**	Co	Cu	-0.07	**223**	Tc	Rh	-0.18	**287**	Cd	Ir	-0.23
**38**	Ti	Rh	0.08	**141**	Co	Tc	0.23	**225**	Tc	Ag	-0.25	**288**	Cd	Pt	-0.22
**39**	Ti	Pd	0.12	**148**	Co	Ta	0.17	**242**	Ru	W	0.09	**295**	Hf	Ir	0.18
**41**	Ti	Cd	0.17	**151**	Co	Os	-0.06	**251**	Rh	Cd	0.04	**323**	Pt	Pt	-0.29
**42**	Ti	Hf	-0.23	**174**	Cu	Zn	0.28	**252**	Rh	Hf	0.28				
**43**	Ti	Ta	-0.28	**179**	Cu	Pd	-0.01	**253**	Rh	Ta	-0.22				

Table S5 limiting potential of H_2 formation for screened catalysts.

ID	M1	M2	$U_L(H_2)$	ID	M1	M2	$U_L(H_2)$	ID	M1	M2	$U_L(H_2)$	ID	M1	M2	$U_L(H_2)$
**12**	Sc	Tc	-0.66	**44**	Ti	W	-0.20	**180**	Cu	Ag	-0.31	**258**	Rh	Pt	-0.07
**14**	Sc	Rh	-0.11	**76**	Cr	Co	-0.42	**188**	Cu	Pt	-1.02	**260**	Pd	Pd	-0.81
**19**	Sc	Ta	-0.06	**96**	Mn	Fe	-0.47	**192**	Zn	Tc	-0.22	**269**	Pd	Pt	-0.81
**26**	Ti	Ti	-0.09	**98**	Mn	Ni	-0.84	**202**	Zn	Os	-0.02	**282**	Cd	Hf	-0.10
**31**	Ti	Co	-1.00	**107**	Mn	Cd	-0.22	**207**	Mo	Tc	-0.57	**283**	Cd	Ta	-0.53
**33**	Ti	Cu	-0.38	**119**	Fe	Cu	-0.46	**212**	Mo	Cd	-0.22	**286**	Cd	Os	-0.21
**34**	Ti	Zn	-0.37	**138**	Co	Cu	-0.32	**223**	Tc	Rh	-0.67	**287**	Cd	Ir	-0.54
**38**	Ti	Rh	-0.43	**141**	Co	Tc	-0.21	**225**	Tc	Ag	-0.38	**288**	Cd	Pt	-0.89
**39**	Ti	Pd	-0.44	**148**	Co	Ta	-2.34	**242**	Ru	W	-0.03	**295**	Hf	Ir	-0.08
**41**	Ti	Cd	-0.03	**151**	Co	Os	-0.17	**251**	Rh	Cd	-0.75	**323**	Pt	Pt	-0.80
**42**	Ti	Hf	-0.24	**174**	Cu	Zn	-0.31	**252**	Rh	Hf	-0.35				
**43**	Ti	Ta	-0.06	**179**	Cu	Pd	-0.95	**253**	Rh	Ta	-0.74				

Table S6 List of 37 DMSCs with limiting potential of H_2 formation lower than -0.1 V.

ID	M1	M2	$U_L(H_2)$	ID	M1	M2	$U_L(H_2)$	ID	M1	M2	$U_L(H_2)$	ID	M1	M2	$U_L(H_2)$
**12**	Sc	Tc	-0.66	**96**	Mn	Fe	-0.47	**180**	Cu	Ag	-0.31	**260**	Pd	Pd	-0.81
**14**	Sc	Rh	-0.11	**98**	Mn	Ni	-0.84	**188**	Cu	Pt	-1.02	**269**	Pd	Pt	-0.81
**31**	Ti	Co	-1.00	**107**	Mn	Cd	-0.22	**192**	Zn	Tc	-0.22	**283**	Cd	Ta	-0.53
**33**	Ti	Cu	-0.38	**119**	Fe	Cu	-0.46	**207**	Mo	Tc	-0.57	**286**	Cd	Os	-0.21
**34**	Ti	Zn	-0.37	**138**	Co	Cu	-0.32	**212**	Mo	Cd	-0.22	**287**	Cd	Ir	-0.54
**38**	Ti	Rh	-0.43	**141**	Co	Tc	-0.21	**223**	Tc	Rh	-0.67	**288**	Cd	Pt	-0.89
**39**	Ti	Pd	-0.44	**148**	Co	Ta	-2.34	**225**	Tc	Ag	-0.38	**323**	Pt	Pt	-0.80

**42**	Ti	Hf	-0.24	**151**	Co	Os	-0.17	**251**	Rh	Cd	-0.75
**44**	Ti	W	-0.20	**174**	Cu	Zn	-0.31	**252**	Rh	Hf	-0.35
**76**	Cr	Co	-0.42	**179**	Cu	Pd	-0.95	**253**	Rh	Ta	-0.74

Table S7 CO_2 adsorption energy for screened catalysts.

**ID**	**M1**	**M2**	$E_{ads}(CO_2)$	**ID**	**M1**	**M2**	$E_{ads}(CO_2)$	**ID**	**M1**	**M2**	$E_{ads}(CO_2)$	**ID**	**M1**	**M2**	$E_{ads}(CO_2)$
**12**	Sc	Tc	-0.03	**96**	Mn	Fe	-0.02	**180**	Cu	Ag	0.33	**260**	Pd	Pd	-0.25
**14**	Sc	Rh	-0.13	**98**	Mn	Ni	-2.05	**188**	Cu	Pt	-0.29	**269**	Pd	Pt	-0.33
**31**	Ti	Co	0.19	**107**	Mn	Cd	-5.17	**192**	Zn	Tc	0.32	**283**	Cd	Ta	0.09
**33**	Ti	Cu	-0.42	**119**	Fe	Cu	0.06	**207**	Mo	Tc	0.27	**286**	Cd	Os	0.32
**34**	Ti	Zn	-0.38	**138**	Co	Cu	0.42	**212**	Mo	Cd	0.11	**287**	Cd	Ir	0.33
**38**	Ti	Rh	-0.03	**141**	Co	Tc	-0.93	**223**	Tc	Rh	0.21	**288**	Cd	Pt	0.17
**39**	Ti	Pd	0.10	**148**	Co	Ta	0.11	**225**	Tc	Ag	0.08	**323**	Pt	Pt	-0.39
**42**	Ti	Hf	-1.12	**151**	Co	Os	-1.50	**251**	Rh	Cd	15.61				
**44**	Ti	W	-0.73	**174**	Cu	Zn	0.01	**252**	Rh	Hf	-0.03				
**76**	Cr	Co	-0.05	**179**	Cu	Pd	-0.22	**253**	Rh	Ta	0.28				

Table S8 List of 19 DMSCs CO_2 adsorption energy for screened catalysts.

**ID**	**M1**	**M2**	$E_{ads}(CO_2)$	**ID**	**M1**	**M2**	$E_{ads}(CO_2)$	**ID**	**M1**	**M2**	$E_{ads}(CO_2)$
**12**	Sc	Tc	-0.03	**76**	Cr	Co	-0.05	**188**	Cu	Pt	-0.29
**14**	Sc	Rh	-0.13	**96**	Mn	Fe	-0.02	**252**	Rh	Hf	-0.03
**33**	Ti	Cu	-0.42	**98**	Mn	Ni	-2.05	**260**	Pd	Pd	-0.25
**34**	Ti	Zn	-0.38	**107**	Mn	Cd	-5.17	**269**	Pd	Pt	-0.33
**38**	Ti	Rh	-0.03	**141**	Co	Tc	-0.93	**323**	Pt	Pt	-0.39
**42**	Ti	Hf	-1.12	**151**	Co	Os	-1.50				
**44**	Ti	W	-0.73	**179**	Cu	Pd	-0.22				

Table S9 The adsorption energies of main products for selected 19 DMSCs. The selected catalysts are marked in red.

**ID**	**M1**	**M2**	**Adsorption Energy**						
			**HCOOH**	CH_3OH	H_2O	CH_4	C_2H_4	C_2H_6	C_2H_6O

**12**	Sc	Tc	-0.08	-0.12	-0.01	0.00	-0.40	-0.27	-0.06
**14**	Sc	Rh	0.10	-0.09	0.11	0.01	-0.26	-0.18	-0.03
**33**	Ti	Cu	-0.26	-0.45	-0.19	0.01	-0.65	-0.25	-0.06
**34**	Ti	Zn	-0.24	-0.44	-0.21	-0.03	-0.66	-0.22	-0.06
**38**	Ti	Rh	-0.36	-0.43	-0.20	0.08	-0.59	-0.20	0.00
**42**	Ti	Hf	-0.84	-0.47	-0.21	0.01	-1.25	-0.27	-0.05
**44**	Ti	W	-0.65	-0.53	-0.26	0.06	-1.19	-0.31	-0.09
**76**	Cr	Co	-0.10	-0.29	-0.03	0.26	-0.64	-0.44	-0.44
**96**	Mn	Fe	-0.08	-0.29	-0.03	-0.21	-0.73	-0.60	-1.10
**98**	Mn	Ni	-3.00	-2.17	1.99	1.51	-2.75	-2.60	-2.39
**107**	Mn	Cd	0.13	0.19	0.30	0.03	-0.14	-0.26	-0.04
**141**	Co	Tc	-1.79	-1.07	0.19	-0.19	-1.85	-1.50	-1.36
**151**	Co	Os	-2.02	-1.47	0.16	-0.04	-2.28	-1.71	-1.64
**179**	Cu	Pd	-0.26	-0.34	-0.14	-0.55	-0.70	-0.81	-0.64
**188**	Cu	Pt	-0.32	-0.43	-0.23	-0.58	-0.80	-0.82	-0.72
**252**	Rh	Hf	0.19	0.16	0.25	0.02	-0.25	-0.24	-0.09
**260**	Pd	Pd	-0.32	-0.41	-0.27	-0.58	-0.83	-0.84	-0.70
**269**	Pd	Pt	-0.43	-0.54	-0.33	-0.68	-0.88	-0.94	-0.79
**323**	Pt	Pt	-0.45	-0.58	-0.41	-0.71	-0.97	-0.99	-0.85

Table S10 Identification of DMSCs through limiting potential of products for DMSCs. The selected catalysts are marked in red. The unites for limiting potential (U_L) and Gibbs free energy change (ΔG) are V and eV, respectively.

ID	M1	M2	$U_L(H_2)$	HCOOH	CH_3OH	CH_4	C_2H_6O
**12**	Sc	Tc	-0.66	U_L = -0.45	—	U_L = -0.45	U_L = -0.45
**14**	Sc	Rh	-0.11		ΔG(OCHO* → HCOOH*) = 0.45<br>ΔG(COOH* → HCOOH*) = 0.47<br>ΔG(CHO* → CHOH*) = 0.71<br>ΔG(COOH* → CO*)		ΔG(COOH* → CO*) = 0.16

						= 0.16	
**33**	Ti	Cu	-0.38	—	—	$\Delta G(CO_2^* \rightarrow COOH^*)$<br>= 0.56<br>$\Delta G(OCHO^* \rightarrow HCOOH^*)$<br>= 1.08	$\Delta G(CO_2^* \rightarrow COOH^*)$<br>= 0.56
**34**	Ti	Zn	-0.37	—	—	$\Delta G(CO_2^* \rightarrow COOH^*)$<br>= 0.56<br>$\Delta G(OCHO^* \rightarrow HCOOH^*)$<br>= 1.05	$\Delta G(CO_2^* \rightarrow COOH^*)$<br>= 0.56
**38**	Ti	Rh	-0.43	—	—	$\Delta G(CO^* \rightarrow COH^*)$<br>= 1.56<br>$\Delta G(CHO^* \rightarrow CHOH^*)$<br>= 1.25	U_L = -0.21
**42**	Ti	Hf	-0.24	—	—	$\Delta G(CO_2^* \rightarrow COOH^*)$<br>= 0.71<br>$\Delta G(OCHO^* \rightarrow HCOOH^*)$<br>= 1.08	$\Delta G(CO_2^* \rightarrow COOH^*)$<br>= 0.71
**44**	Ti	W	-0.2	—	—	$\Delta G(COOH^* \rightarrow CO^*)$<br>= 0.68<br>$\Delta G(COOH^* \rightarrow HCOOH^*)$<br>= 0.45<br>$\Delta G(OCHO^* \rightarrow HCOOH^*)$<br>= 0.77	$\Delta G(COOH^* \rightarrow CO^*)$<br>= 0.68
**76**	Cr	Co	-0.42	—	—	$\Delta G(CO^* \rightarrow COH^*)$<br>= 4.09<br>$\Delta G(CO^* \rightarrow CHO^*)$<br>= 1.31<br>$\Delta G(HCOOH^* \rightarrow CHO^*)$<br>= 1.34	—
**96**	Mn	Fe	-0.47	U_L = -0.3	—	$\Delta G(CO^* \rightarrow CHO^*)$<br>= 1.1<br>$\Delta G(HCOOH^* \rightarrow CHO^*)$	—

						= 0.96	
**98**	Mn	Ni	-0.83	—	—	$\Delta G(CH_3^* \rightarrow CH_4^*)$<br>= 1.58<br>$\Delta G(OH^* \rightarrow H_2O^*)$<br>= 2.59	—
**107**	Mn	Cd	-0.22		$\Delta G(CO_2^* \rightarrow COOH^*)$<br>= 5.7<br>$\Delta G(CO_2^* \rightarrow OCHO^*)$<br>= 6.00		—
**141**	Co	Tc	-0.21	—	—	$\Delta G(COOH^* \rightarrow CO^*)$<br>= 1.16<br>$\Delta G(HCOOH^* \rightarrow CHO^*)$<br>= 2.82	—
**151**	Co	Os	-0.17	—	—	$\Delta G(CO_2^* \rightarrow COOH^*)$<br>= 0.20<br>$\Delta G(CO_2^* \rightarrow OCHO^*)$<br>= 0.98	—
**252**	Rh	Hf	-0.35	U_L = -0.29	U_L = -0.29	$\Delta G(CO^* \rightarrow COH^*)$<br>= 1.47<br>$\Delta G(CHO^* \rightarrow CHOH^*)$<br>= 1.51<br>$\Delta G(CH_2OH^* \rightarrow CH_2^*)$<br>= 0.58	U_L = -0.29

Table S11 Free energies for elementary steps for CO_2RR to HCOOH and CH_4 on Sc/Tc DMSC.

Step Index	Reaction	Reaction Free Energy (eV)
1	$CO_2 + * \rightarrow CO_2^*$	-0.03
2	$CO_2^* + H^+ + e^- \rightarrow COOH^*$	0.45
3	$CO_2^* + H^+ + e^- \rightarrow HCOOH^*$	-0.16
4	$HCOOH^* \rightarrow HCOOH + *$	0.08

5	$HCOOH^* + H^+ + e^- \rightarrow CHO^* + H_2O$	0.14
6	$CHO^* + H^+ + e^- \rightarrow CH_2O^*$	0.03
7	$CH_2O^* + H^+ + e^- \rightarrow CH_3O^*$	-0.99
8	$CH_3O^* + H^+ + e^- \rightarrow CH_3OH^*$	0.33
9	$CH_3OH^* + H^+ + e^- \rightarrow OH^* + CH_4$	-1.30
10	$OH^* + H^+ + e^- \rightarrow H_2O^*$	0.15
11	$H_2O^* \rightarrow H_2O + *$	0.01

Table S12 Free energies for elementary steps for CO_2RR to HCOOH on Mn/Fe DMSC.

Step Index	Reaction	Reaction Free Energy (eV)
1	$CO_2 + * \rightarrow CO_2^*$	-0.02
2	$CO_2^* + H^+ + e^- \rightarrow OCHO^*$	0.30
3	$OCHO^* + H^+ + e^- \rightarrow HCOOH^*$	-0.02
4	$HCOOH^* \rightarrow HCOOH + *$	0.08

Table S13 Free energies for elementary steps for CO_2RR to HCOOH and CH_3OH on Rh/Hf DMSC.

Step Index	Reaction	Reaction Free Energy (eV)
1	$CO_2 + * \rightarrow CO_2^*$	-0.03
2	$CO_2^* + H^+ + e^- \rightarrow COOH^*$	0.29
3	$COOH^* + H^+ + e^- \rightarrow HCOOH^*$	0.27
4	$HCOOH^* \rightarrow HCOOH + *$	-0.19
5	$HCOOH^* + H^+ + e^- \rightarrow CHO^* + H_2O$	-0.45
6	$CHO^* + H^+ + e^- \rightarrow CH_2O^*$	0.20
7	$OCH_2^* + H^+ + e^- \rightarrow CH_2OH^*$	-0.06
8	$CH_2OH^* + H^+ + e^- \rightarrow CH_3OH^*$	-0.16
9	$CH_3OH^* \rightarrow CH_3OH + *$	-0.16

Table S14 Free energies for elementary steps for CO_2RR to CH_3CH_2OH on Sc/Tc DMSC.

Step Index	Reaction	Reaction Free Energy (eV)

1	$CO_2 + * \rightarrow CO_2*$	-0.03
2	$CO_2* + H^+ + e^- \rightarrow COOH*$	0.45
3	$COOH* + H^+ + e^- \rightarrow CO* + H_2O$	-0.07
4	$2CO* \rightarrow CO\text{-}CO* + *$	-0.77
5	$CO\text{-}CO* + H^+ + e^- \rightarrow CO\text{-}COH*$	0.33
6	$CO* + H^+ + e^- \rightarrow CHO*$	0.05
7	$CO* + CHO* \rightarrow CO\text{-}CHO* + *$	-0.26
8	$CO\text{-}CHO* + H^+ + e^- \rightarrow CHO\text{-}COH*$	-0.07
9	$CO\text{-}COH* + H^+ + e^- \rightarrow CHO\text{-}COH*$	0.16
10	$CHO\text{-}COH* + H^+ + e^- \rightarrow CHOH\text{-}COH*$	0.19
11	$CHOH\text{-}COH* + H^+ + e^- \rightarrow CH\text{-}COH* + H_2O$	-0.45
12	$CH\text{-}COH* + H^+ + e^- \rightarrow CH_2\text{-}COH*$	-0.50
13	$CH_2\text{-}COH* + H^+ + e^- \rightarrow CH_2\text{-}CHOH*$	-0.39
14	$CH_2\text{-}CHOH* + H^+ + e^- \rightarrow CH_2\text{-}CH_2OH*$	0.38
15	$CH_2\text{-}CH_2OH* + H^+ + e^- \rightarrow CH_3\text{-}CH_2OH*$	-0.73
16	$CH_3\text{-}CH_2OH* \rightarrow CH_3\text{-}CH_2OH + *$	0.06

Table S15 Free energies for elementary steps for CO_2RR to CH_3CH_2OH on Ti/Rh DMSC.

Step Index	Reaction	Reaction Free Energy (eV)
1	$CO_2 + * \rightarrow CO_2*$	-0.03
2	$CO_2* + H^+ + e^- \rightarrow COOH*$	0.21
3	$COOH* + H^+ + e^- \rightarrow CO* + H_2O$	0.10
4	$2CO* \rightarrow CO\text{-}CO* + *$	0.20
5	$CO\text{-}CO* + H^+ + e^- \rightarrow CO\text{-}COH*$	0.12
6	$CO\text{-}COH* + H^+ + e^- \rightarrow CHO\text{-}COH*$	-0.31
7	$CHO\text{-}COH* + H^+ + e^- \rightarrow CHOH\text{-}COH*$	0.19
8	$CHOH\text{-}COH* + H^+ + e^- \rightarrow CH\text{-}COH* + H_2O$	-0.56
9	$CH\text{-}COH* + H^+ + e^- \rightarrow CH\text{-}CHOH*$	-0.79
10	$CH\text{-}CHOH* + H^+ + e^- \rightarrow CH_2\text{-}CHOH*$	-0.28
11	$CH_2\text{-}CHOH* + H^+ + e^- \rightarrow CH_2\text{-}CH_2OH*$	0.19
12	$CH_2\text{-}CH_2OH* + H^+ + e^- \rightarrow CH_3\text{-}CH_2OH*$	-0.32

13	CH_3-CH_2OH^* → CH_3-CH_2OH + *	0.00

Table S16 Free energies for elementary steps for CO_2RR to CH_3CH_2OH on Rh/Hf DMSC.

Step Index	Reaction	Reaction Free Energy (eV)
1	CO_2 + * → CO_2^*	-0.03
2	CO_2^* + H^+ + e^- → COOH*	0.29
3	COOH* + H^+ + e^- → CO* + H_2O	0.22
4	2CO* → CO-CO* + *	0.33
5	CO-CO* + H^+ + e^- → CO-CHO*	-0.91
6	CO* + H^+ + e^- → CHO*	-0.40
7	CO* + CHO* → CO-CHO* + *	-0.19
8	CO-CHO* + H^+ + e^- → CHO-CHO*	-0.11
9	CHO-CHO* + H^+ + e^- → CHO-CHOH*	-0.03
10	CHO-CHOH* + H^+ + e^- → CH-CHO* + H_2O	0.06
11	CH-CHO* + H^+ + e^- → CH_2-CHO*	-1.04
12	CH_2-CHO* + H^+ + e^- → CH_2-CHOH*	0.10
13	CH_2-CHOH* + H^+ + e^- → CH_2-CH_2OH^*	-0.10
14	CH_2-CH_2OH^* + H^+ + e^- → CH_3-CH_2OH^*	-0.36
15	CH_3-CH_2OH^* → CH_3-CH_2OH + *	0.09

Table S16 Bader charge transfer of C, N, O, H and central metal atoms on Cu/Cu and Sc/Tc DMSCs.

Bader charge (|e|)	Cu/Cu	Cu/Cu-CO_2	Cu/Cu-H	Bader charge (|e|)	Sc/Tc	Sc/Tc-CO_2	Sc/Tc-H
**C**	—	-2.11	—	**C**	—	-1.42	—
**N1**	1.22	1.23	1.24	**N1**	1.23	1.29	1.23
**N2**	1.14	1.10	1.15	**N2**	1.17	1.16	1.16
**N3**	1.24	1.24	1.25	**N3**	1.12	1.11	1.19
**N4**	1.29	1.29	1.30	**N4**	1.23	1.17	1.23
**N5**	1.06	1.14	1.12	**N5**	1.14	1.11	1.14
**N6**	1.33	1.32	1.34	**N6**	1.17	1.17	1.19
**Cu1**	-0.97	-0.95	-0.94	**Sc**	-1.81	-1.83	-1.78

**Cu2**	-0.96	-0.95	-0.94	**Tc**	-1.03	-1.36	-1.30
**O1**	—	1.07	—	**O1**	—	1.14	—
**O2**	—	1.06	—	**O2**	—	1.18	—
**H**	—	—	-0.48	**H**	—	—	0.20

Table S17 Bond center of Cu/Cu and Sc/Tc DMSCs.

Cu/Cu	Bond center (eV)	Sc/Tc	Bond center (eV)
Cu1	-3.1	Sc	1.9
Cu2	-3.1	Tc	-1.2
N1	-3.9	N1	-3.6
N2	-2.8	N2	-3.0
N3	-3.9	N3	-3.9
N4	-3.9	N4	-3.6
N5	-2.8	N5	-3.0
N6	-3.9	N6	-4.0

Reference

(1) Bai, Z.; Jiang, X. Z.; Luo, K. H. Theoretical exploration on the performance of single and dual-atom Cu catalysts on the CO 2 electroreduction process: a DFT study. *Physical Chemistry Chemical Physics* **2023**, *25* (35), 23717-23727.
